# Internal-state-dependent control of feeding behavior via hippocampal ghrelin signaling

**DOI:** 10.1016/j.neuron.2023.10.016

**Published:** 2024-01-17

**Authors:** Ryan W.S. Wee, Karyna Mishchanchuk, Rawan AlSubaie, Timothy W. Church, Matthew G. Gold, Andrew F. MacAskill

**Affiliations:** 1 Department of Neuroscience, Physiology and Pharmacology, University College London, Gower St., London WC1E 6BT, UK

**Keywords:** hunger, hippocampus, ghrelin, synaptic inhibition, nucleus accumbens, internal state, feeding behavior

## Abstract

Hunger is an internal state that not only invigorates feeding but also acts as a contextual cue for higher-order control of anticipatory feeding-related behavior. The ventral hippocampus is crucial for differentiating optimal behavior across contexts, but how internal contexts such as hunger influence hippocampal circuitry is unknown. In this study, we investigated the role of the ventral hippocampus during feeding behavior across different states of hunger in mice. We found that activity of a unique subpopulation of neurons that project to the nucleus accumbens (vS-NAc neurons) increased when animals investigated food, and this activity inhibited the transition to begin eating. Increases in the level of the peripheral hunger hormone ghrelin reduced vS-NAc activity during this anticipatory phase of feeding via ghrelin-receptor-dependent increases in postsynaptic inhibition and promoted the initiation of eating. Together, these experiments define a ghrelin-sensitive hippocampal circuit that informs the decision to eat based on internal state.

## Highlights

• Ventral hippocampal neurons are active during investigation of food
• Activity in neurons that project to the nucleus accumbens inhibits initiation of eating
• Peripheral ghrelin reduces this activity and promotes initiation of eating
• Via ghrelin-receptor-dependent increases in postsynaptic inhibition

## Teaser

Wee et al. show that ventral hippocampal neurons that project to nucleus accumbens are active around investigation of food, and this activity inhibits the initiation of eating. Increases in the level of the peripheral hunger hormone ghrelin reduce this activity and promote eating via ghrelin-receptor-dependent increases in postsynaptic inhibition.

## Introduction

Animals must be able to control feeding behavior dependent on need. Consuming food when already sated utilizes time and energy that could be spent on more essential functions and can result in disease and disorders associated with overeating. In contrast, being unable to sense the need for food—or “hunger”—can result in undereating and the resultant lack of fitness.^1^

Although feeding behavior can be conceptualized as a problem of optimal control, where deviations from a caloric set-point motivate behavior to correct the deviation,^2^^,^^3^ animals often instead anticipate future changes in their hunger state to produce behaviors well in advance of shifts in caloric balance.^3^^,^^4^ This predictive aspect of homeostatic regulation is increasingly recognized to be a crucial determinant of goal-directed behavior that defines feeding,^5^^,^^6^^,^^7^ where food-associated stimuli are assigned value via Pavlovian and instrumental learning.^7^^,^^8^ Such value-based associations between sensory cues and the consumption of food are therefore crucial for efficient anticipatory behavior around food.^7^^,^^9^^,^^10^

A key aspect of this process is the ability to integrate external cues with an internal state such as hunger.^1^^,^^11^^,^^12^ This is because the value of a given food cue is ambiguous—the same food would predict a rewarding post-ingestive outcome when the animal is hungry, but not when the animal is sated.^13^ In this framework, hunger must act as a context upon which the optimal behavior toward sensory cues is interpreted.^13^^,^^14^^,^^15^^,^^16^^,^^17^^,^^18^

The hippocampus has been repeatedly proposed as a crucial structure for defining behavior dependent on context, most notably in spatial contextual associations.^19^^,^^20^^,^^21^^,^^22^^,^^23^ Interestingly, there is a proposed dichotomy between dorsal and ventral hippocampal circuitry, with dorsal circuitry being classically associated with the dissociation of events based on their spatial and temporal context and ventral circuits being more strongly associated with context-specific, goal directed, and affective behavior.^21^^,^^22^^,^^23^^,^^24^^,^^25^^,^^26^^,^^27^^,^^28^^,^^29^ In particular, neurons in the ventral CA1/subiculum (vS) area of the ventral hippocampus are proposed to be key for supporting affective behavior based on contextual information.^30^^,^^31^^,^^32^^,^^33^^,^^34^^,^^35^

The hippocampus is also heavily involved in hunger sensing in both humans and rodents.^13^^,^^14^^,^^15^^,^^16^^,^^36^^,^^37^ This suggests that, in addition to spatial context, the hippocampus may also differentiate behavior dependent on other, more abstract contexts such as hunger. Consistent with this idea, hippocampal activity is extremely sensitive to hunger state in both humans^36^^,^^37^ and rodents,^38^^,^^39^^,^^40^ and inactivation and dysfunction of the hippocampus leads to impaired hunger-based decision making.^14^^,^^15^^,^^16^^,^^41^^,^^42^^,^^43^ Moreover, the hippocampus expresses the receptor for the peripheral hunger hormone ghrelin (GHSR1a) in both rodents^44^^,^^45^^,^^46^^,^^47^^,^^48^^,^^49^ and non-human primates.^50^ Interestingly, peripherally circulating hormones are able to gain access to the hippocampus,^51^ and there is evidence to support the entry of peripheral ghrelin into the hippocampus through the blood-brain barrier (BBB)^48^^,^^52^ (but see Furness et al.^53^). Once present in the hippocampus, ghrelin is capable of not only inducing structural and functional plasticity^48^^,^^54^ but also influencing anticipatory behavior and choice.^47^^,^^48^^,^^55^^,^^56^

However, while it is clear that the motivational state affects hippocampal processing, and hippocampal dysfunction impairs hunger-dependent behavior, how hippocampal circuitry directly influences internal-state-dependent feeding behavior, and the cellular and circuit mechanisms underlying this ability, remains unknown. This is compounded by the fact that the vS is composed of multiple, non-overlapping and functionally distinct parallel projections to distinct downstream areas.^57^^,^^58^^,^^59^^,^^60^^,^^61^^,^^62^ For example, neurons in the vS that project to the nucleus accumbens (NAc) have been shown to preferentially represent and control motivation and value,^31^^,^^32^^,^^33^^,^^58^ to be preferentially active during anticipatory behavior,^34^ and inhibited upon eating,^63^ reminiscent of a much-hypothesized role in behavioral inhibition,^64^ where increased activity of vS-NAc neurons inhibits non-optimal ongoing behavior.^63^^,^^65^ Similarly, a separate population of neurons projecting to the lateral hypothalamus (LH) has been shown to be recruited during salient environments and during learning of food associations.^14^^,^^35^^,^^47^ Both of these populations of neurons are therefore well placed to control anticipatory feeding-related behavior.^14^^,^^31^^,^^32^^,^^33^^,^^35^^,^^47^^,^^58^^,^^63^ However, how these populations are uniquely used during feeding behavior and how they are influenced by internal state signaled by peripheral ghrelin is unknown.

Together, the vS is well placed to control anticipatory feeding-related behavior. It is consistently implicated in hunger-based decisions, its dysfunction impairs behavior requiring hunger sensing, and it has ghrelin-sensitive neurons that project to two brain regions both crucially important for defining feeding behavior. Therefore, in this study, we used a combination of quantitative behavior, *in vivo* imaging and manipulation, and slice physiology to address the role of vS circuitry in hunger-based decisions.

## Results

### Peripheral ghrelin administration increases the transition from food investigation to food consumption

Feeding behavior can be described as the chaining together of distinct, stereotyped behaviors, such as exploratory sniffing and investigation of food (presented here as “Inv”), food consumption (“Eat”), as well as non-feeding behaviors such as “Rear,” “Groom,” and “Rest” (“Oth”).^66^ Increases in peripheral ghrelin are known to dramatically alter behavior toward food, in particular through the promotion of the initiation of eating. However, despite intensive investigation, it is unclear how increases in peripheral ghrelin alter the structure of this moment-to-moment behavior around food.

**Figure 1:**
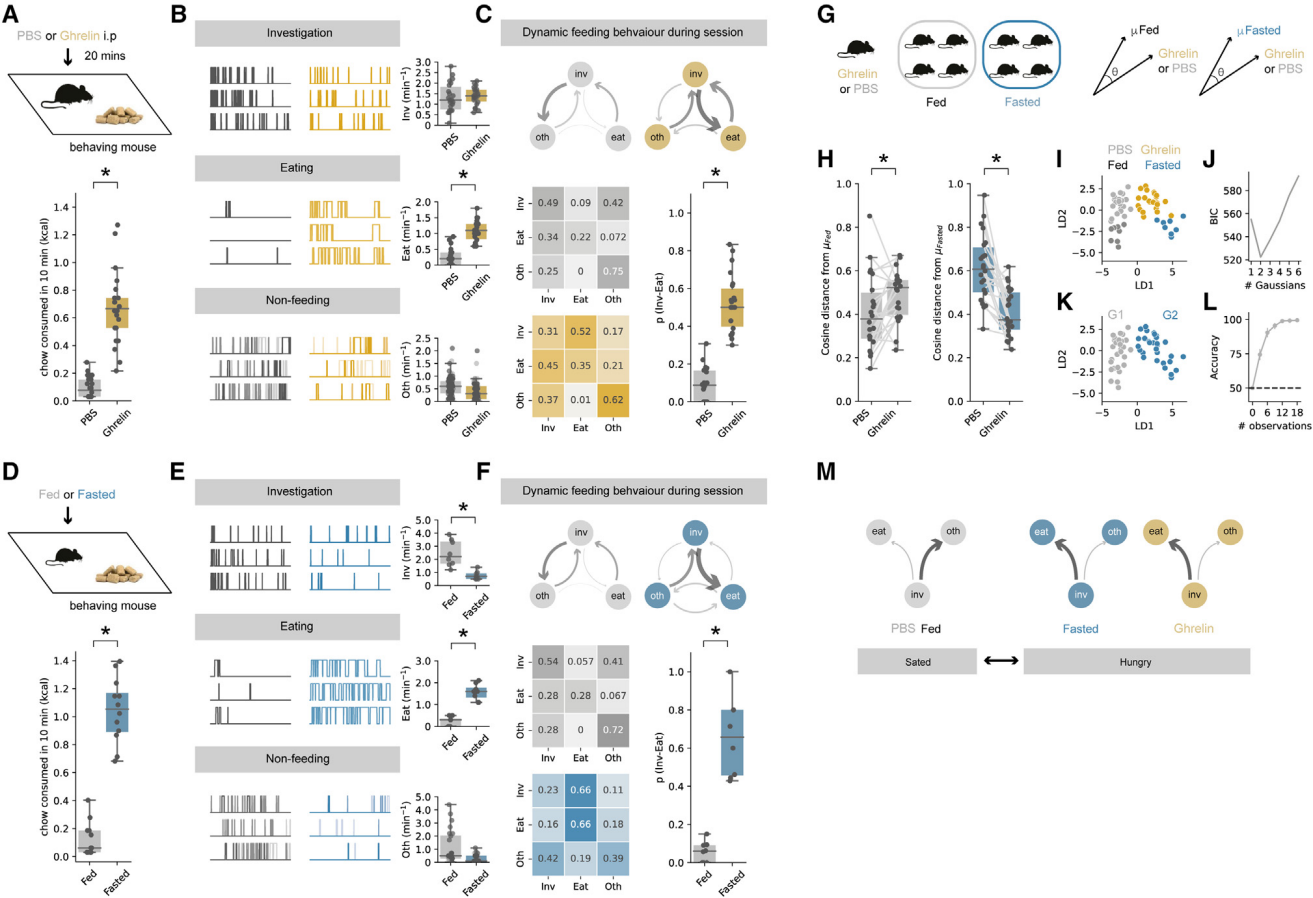
Peripheral ghrelin administration increases the transition from food investigation to food consumption (A) Top: schematic of experiment. Bottom: ghrelin administration (gold) increases chow consumption compared with PBS control (gray, n = 22 mice). (B) Analysis of food investigation, eating, and non-feeding behaviors, including grooming, quiet resting, and rearing. Plots show three examples of mouse behavior across example 10-min sessions. (C) Markov analysis of feeding behavior during 10-min session. Top: state transitions for PBS (gray) and ghrelin-treated (gold) mice. Arrow thickness is proportional to the probability of transition. Bottom, left: transition matrix for PBS- and ghrelin-treated mice. Bottom, right: summary of investigation to eat transition across all mice in PBS and ghrelin. (D–F) As in (A)–(C) but comparing *ad-libitum*-fed mice (gray) with overnight-fasted mice (blue, n = 8 mice). (G) Strategy for cosine similarity analysis. (H) Cosine distance of PBS- and ghrelin-treated mice compared with fed (gray, left), and fasted (blue, right) mice. (I) Fed, fasted, PBS-treated, and ghrelin-treated behavior across the first two dimensions (LD1 and LD2) after linear discriminant analysis (LDA). (J) Bayesian information criterion (BIC) scores of fits to LDA distributions with increasing numbers of Gaussians. (K) Mapping of best fitting clusters. (L) Robustness of clustering shown by accuracy of random forest classifiers trained on increasing subsets of the clustered data. (M) Schematic of behavioral problem. Boxplots represent the median, 75^th^, and 95^th^ percentiles, and individual datapoints are superimposed for clarity.

To address this, we first confirmed that ghrelin injections caused an increase in food consumption in sated mice when they were repeatedly presented with a familiar food item (a chow pellet) in a well-habituated arena, when compared with the injection of PBS vehicle control (Figure 1A). Next, by scoring each behavior performed by the mouse during the session, we found that this increased consumption resulted from a large and specific increase in the frequency of initiating eating, with only minimal change in the frequency of food investigation or the frequency of non-feeding behaviors (Figure 1B).

Next, we asked how sequences of behavior changed within each session to result in this increase in eating. To quantitatively describe the organization of such sequences, we analyzed scored behavior (Inv, Eat, and Oth) as a discrete-time Markov chain—a vector of behavioral states that change as a function of time.^12^ We then computed the transition matrix P*ij* for each animal, which defines the animal’s probability of transitioning from behavior *i* to behavior *j* during the session, and compared these matrices across different states of hunger (Figure 1C).

Using this analysis, we found that the effect of ghrelin was very specific and was centered around transitions from food investigation (Inv). Although PBS-treated mice frequently investigated the food pellet, this investigation was very rarely followed by a transition to eating. In contrast, in ghrelin-treated mice, the frequency of investigation of the food pellet was not changed (Figures 1B and S1), but the transition from investigation to eating was substantially increased. Overall, this suggests that the effect of ghrelin was to increase the probability of transitioning from investigation of food to consumption of food (p_(Inv→Eat)_), with only minimal effect on other behavioral transitions (Figures 1C and S1). This specific alteration in behavior can account for previous work suggesting a key role of ghrelin signaling in the anticipation and initiation of feeding.

### Ghrelin administration influences behavior in a manner similar to natural hunger

The level of peripheral ghrelin is only one of many complex and interacting factors that control behavior around food.^9^ Therefore, we next asked to what extent these changes to behavior upon ghrelin administration were similar to changes in behavior elicited by natural hunger. To do this, we repeated our behavioral assay but instead compared mice that had *ad libitum* access to food (“fed”), to those where food had been removed overnight (“fasted”). We first confirmed that overnight fasting increased chow consumption to levels similar to those of peripheral ghrelin administration (Figure 1D). Next, we constructed Markov chains to demonstrate that, similar to ghrelin administration, overnight fasting resulted in a marked increase in the probability of transitioning from investigation to consumption of food (Figures 1E and 1F). In fasted animals, this was accompanied by an increase in the probability of transitioning from eating directly back to eating, suggesting additional changes to behavior not present in animals treated with ghrelin alone. Together, this qualitatively suggests that the influence of ghrelin on initiation of eating is similar to that induced by natural fasting.

We next wanted to compare mouse behavior after ghrelin administration and fasting more quantitatively. We did this in two ways. First, we measured the cosine distance (a measure of dissimilarity) between the behavioral matrices (Figure 1G). Consistent with our qualitative analysis, PBS-treated mice were more similar (had a lower cosine distance) to fed mice compared with fasted mice, while ghrelin-treated mice were more similar to fasted mice compared with fed mice (Figure 1H). Second, we used linear discriminant analysis (LDA) to find a linear combination of features that optimally separated the behavior of fed, fasted, PBS-treated, and ghrelin-treated mice (Figure 1I). We then used Gaussian mixture modeling to show that this distribution was best described by 2 clusters (Figure 1J), which corresponded to fed plus PBS-treated and fasted plus ghrelin-treated, respectively (Figure 1K). Finally, we tested the robustness of this clustering by showing that random forest classifiers trained on a small subset of this dataset (e.g., as low as 6 out of a total of 60 datapoints, or 10%) could classify with an accuracy of >90% (Figure 1L).

Together, this analysis suggests that both peripheral ghrelin administration and natural fasting result in a change in the probability of transitioning from investigation to consumption of food. This problem can be characterized as one of conditional ambiguity,^13^^,^^17^ where the appropriate behavior toward the same food-associated cue is different, dependent on the internal state (Figure 1M). When hungry (in either ghrelin or fasted conditions), interaction with food cues during investigation frequently results in a transition to consumption, but when sated (in either PBS-treated of fed conditions), interaction with the same food cues instead results in a transition away from consumption.

### vS-NAc activity tracks investigation and eating behavior

One brain area that is thought to play a key role in the resolution of conditional ambiguity is the ventral hippocampus,^13^^,^^17^ via its strong excitatory projections from the vS to downstream areas such as the NAc^31^^,^^32^^,^^33^^,^^57^^,^^58^ and the LH,^14^^,^^35^^,^^47^^,^^57^ which arise from distinct, minimally overlapping populations of neurons.^57^^,^^62^^,^^67^ Therefore, we next investigated (1) how activity in the vS was modulated during feeding behavior and (2) how this activity was distributed across the two populations of projection neurons.

**Figure 2:**
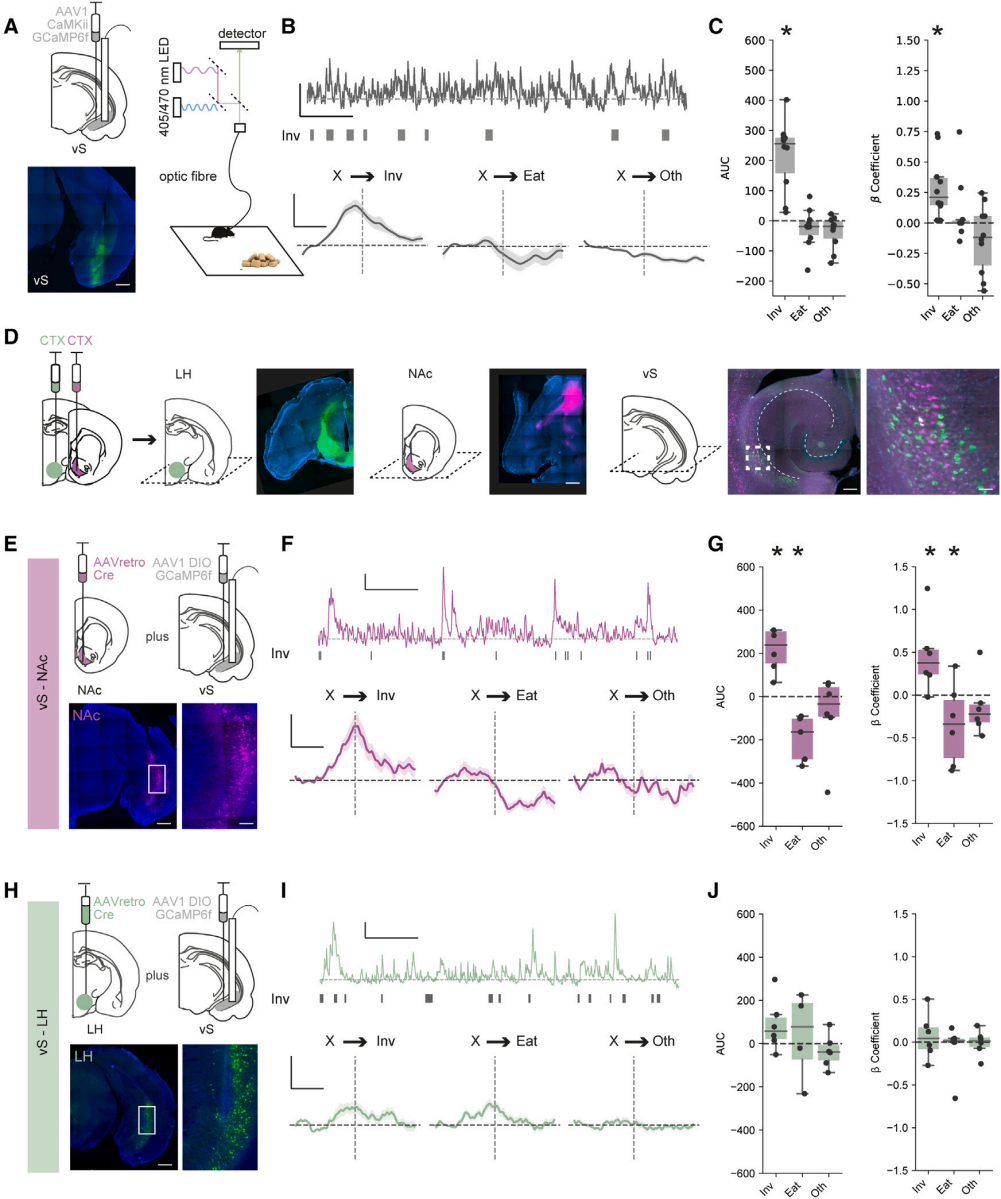
vS neurons that project to the NAc are active during investigation of food (A) Left: schematic (top) and example (bottom) of injection of GCaMP6f and implantation of optical fiber in the vS. Right: photometry setup to allow bulk imaging during free behavior. Scale bars, 1 mm. (B) Top: example photometry trace from vS neurons during the session, with start point of investigative (Inv) behavioral events plotted as raster plots below. Scale bars, 1 *Z* scored fluorescence (zF), 2 min. Bottom: average activity for vS neurons across all mice aligned to start of behavioral events during the session. Scale bars, 0.5 zF, 4 s. (C) Summary of activity around each behavior for vS neurons, summarized using either the area under the curve (AUC) of event-aligned activity (left) or using the coefficients of a generalized linear model fit to the calcium data (right, see STAR Methods). n = 10 mice. (D) Retrograde labeling of vS projections to the LH and NAc, identified by injections of cholera toxin (CTX). Left: injection in LH (green) and NAc (purple) shown in horizontal slices. Right: retrogradely labeled neurons in the vS. Scale bars: top, 500 μm; bottom, 200 μm; 25 μm zoom. (E) Top: schematic of injections allowing intersectional targeting of vS-NAc neurons for photometry. Bottom: example images of neurons in the vS. Scale bars, 500 μm; 100 μm zoom. (F and G) As in (B) and (C) but for vS-NAc neurons in n = 6 mice. (H–J) As in (E)–(G) but for vS-LH neurons in n = 6 mice. Boxplots represent the median, 75^th^, and 95^th^ percentiles, and individual datapoints are superimposed for clarity.

We first recorded bulk calcium activity from excitatory neurons in the vS as sated mice were freely behaving in response to the presentation of chow, as described above (Figure 2A). For each mouse, we then aligned this calcium activity to the onset of each class of behavior (either Inv, Eat, or Oth; Figure 2B). We noticed that there was a large and consistent increase in the activity of vS neurons leading up to and during the investigation of food, reminiscent of previous descriptions of these neurons ramping toward salient locations^34^^,^^68^ (Figures 2B and 2C). This suggests that, consistent with a role during the conditional ambiguity surrounding the transition from investigation to consumption, vS neurons are active during anticipation and investigation of food.

We next asked how this activity was distributed across the two populations of projection neurons (vS-NAc and vS-LH). We first confirmed that these two projections were composed of largely non-overlapping populations (Figures 2D and S2A–S2E). Similar to previous results,^57^ despite being intermingled within the vS, we found only limited overlap between these two populations, suggesting that they form two distinct populations of neurons.

Next, we recorded bulk calcium activity of each projection pathway as mice were freely behaving in response to the presentation of chow (Figures 2E–2I). For each mouse, we again aligned this calcium activity to the onset of each class of behavior (either Inv, Eat, or Oth; Figure 2D). Consistent with our recordings of overall vS activity, we noticed that there was a large and consistent increase in the activity of vS-NAc neurons leading up to and during the investigation of food (Figures 2F and 2G). In addition, there was a consistent drop in vS-NAc activity upon the initiation of eating.^63^ Together, this suggests that vS-NAc neurons are active during anticipation and investigation of food but are then rapidly inhibited upon the commencement of feeding. In contrast, despite multiple suggestions of a crucial role for vS-LH neurons in learning about food-dependent and other affective cues, in this simple assay vS-LH neurons did not show any consistent activity that was time locked to exploratory or feeding behavior in this task (Figures 2I and 2J). Instead vS-LH neurons showed robust activity in response to salient stimuli, such as the presentation of an object or chow^69^ (Figures S2F and S2G). Together, this suggests that the activity of vS-NAc neurons is bidirectionally modulated during investigation and consumption of familiar food. Moreover, increases in vS-NAc activity around the investigation of food is consistent with a role in the anticipation of feeding.

As vS-NAc activity was concentrated around investigation and our behavioral analysis had identified this as a key transition in defining eating behavior (Figure 1), we next wanted to test whether the level of vS-NAc activity during food investigation might define the decision to transition to eat on a moment-to-moment basis. To investigate whether this was the case, we compared activity around investigative bouts followed by eating and those not followed by eating (Figure S2H). Interestingly, vS-NAc activity was constant during investigation in both conditions (Figures S2I and S2J). This suggests that, contrary to vS-NAc activity reflecting the moment-to-moment decision to transition from investigation to eating, it instead may reflect a more stable, long-term signal across multiple investigative bouts, which we reasoned may be related to the internal state of the animal.

Finally, as activity around food in other brain regions such as the hypothalamus is often dependent on the edibility or palatability of the item being investigated,^5^^,^^70^^,^^71^ we asked whether vS activity was similarly sensitive to edibility and palatability. We repeated our imaging experiment but presented either a well-habituated non-food object (a universal tube lid) or highly palatable peanut butter in place of chow. In both of these conditions, we again saw similar ramping activity in vS-NAc neurons during investigation (Figure S2K). This suggests that vS-NAc activity around investigation is present during investigation of salient, non-food objects as well as chow and also during investigation of more palatable food such as peanut butter (Figure S2L).

### vS-NAc activity during food investigation is inhibited by ghrelin

Our previous results revealed that there was a large anticipatory ramp-up of activity in vS-NAc neurons during investigation of food (Figure 2). As the effect of ghrelin was to alter the consequences of such investigative behavior (Figure 1), we next wanted to investigate how this activity in the vS was modulated by increases in peripheral ghrelin.

We first repeated our investigation of overall vS activity in mice with counterbalanced injections of either ghrelin or PBS (Figures S3A–S3D). We found that ghrelin administration resulted in a marked decrease in activity during investigation of chow when compared with PBS-treated mice (Figure S3D). In addition, consistent with the behavioral similarity of ghrelin administration and natural fasting, we found a similar decrease in vS activity when mice were fasted overnight (Figures S3E and S3F). Together, these data suggest that vS activity around investigation is reduced by ghrelin administration as well as natural hunger.

**Figure 3:**
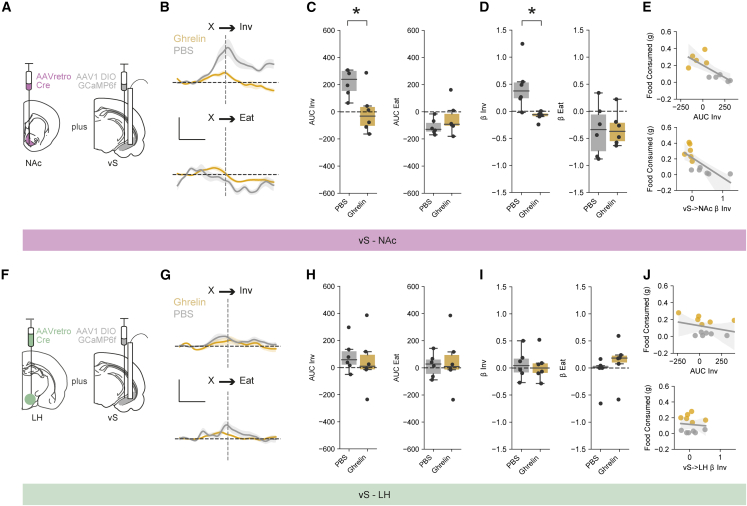
Increased peripheral ghrelin inhibits vS-NAc activity during food investigation (A) Schematic of injections allowing intersectional targeting of vS-NAc neurons for photometry. (B) Top: average activity for vS-NAc neurons across all mice aligned to investigation, after injection of either PBS (gray) or ghrelin (gold). Bottom: average activity around eating. Scale bars, 0.5 zF, 4 s. (C and D) Summary of activity around investigation (left) and eating (right) for vS-NAc neurons after injection of either PBS (gray) or ghrelin (gold). (C) shows event-aligned AUC, (D) shows coefficients of a generalized linear model, n = 6 mice. (E) Correlation between vS-NAc activity during investigation and chow consumption, using AUC (top) or coefficients (bottom). (F–J) As in (A)–(E) but for vS-LH neurons in n = 6 mice. Boxplots represent the median, 75^th^, and 95^th^ percentiles, and individual datapoints are superimposed for clarity.

As our previous experiments found that activity around investigation was specific to vS-NAc neurons, we next repeated our ghrelin manipulations while specifically recording activity of vS-NAc or vS-LH neurons. We first ensured that ghrelin administration had a similar behavioral effect on both cohorts of mice, and found that ghrelin injections increased both total consumption of chow, but also specifically the transition from investigation to eating in both cohorts of mice (Figures S3G–S3J). Importantly, however, this change in behavior was accompanied by an almost complete reduction in the activity of vS-NAc neurons during food investigation (Figures 3B–3E). This effect seemed to be specific to activity around the investigation of food, as: (1) alterations in vS-NAc activity upon eating (Figures 3B–3D) and non-feeding events, such as the presentation of chow and rearing (Figures S3Q–S3S), were maintained across both ghrelin- and PBS-treated animals in the same recordings; (2) there was limited effect of ghrelin on vS-NAc activity during equivalent investigation of a non-food object (Figures S3K–S3M); and (3) ghrelin administration alone had no effect on tonic vS-NAc activity (Figures S3N–S3P).

Interestingly, consistent with the lack of activity around feeding-related behaviors in vS-LH neurons at baseline, there was no effect of ghrelin on vS-LH neurons and activity remained invariant across each behavior (Figures 3F–3J and S3G–S3U), suggesting a projection-specific modulation of vS neurons upon increases of peripheral ghrelin.

### Increasing peripheral ghrelin increases inhibitory postsynaptic amplitude in vS-NAc neurons

**Figure 4:**
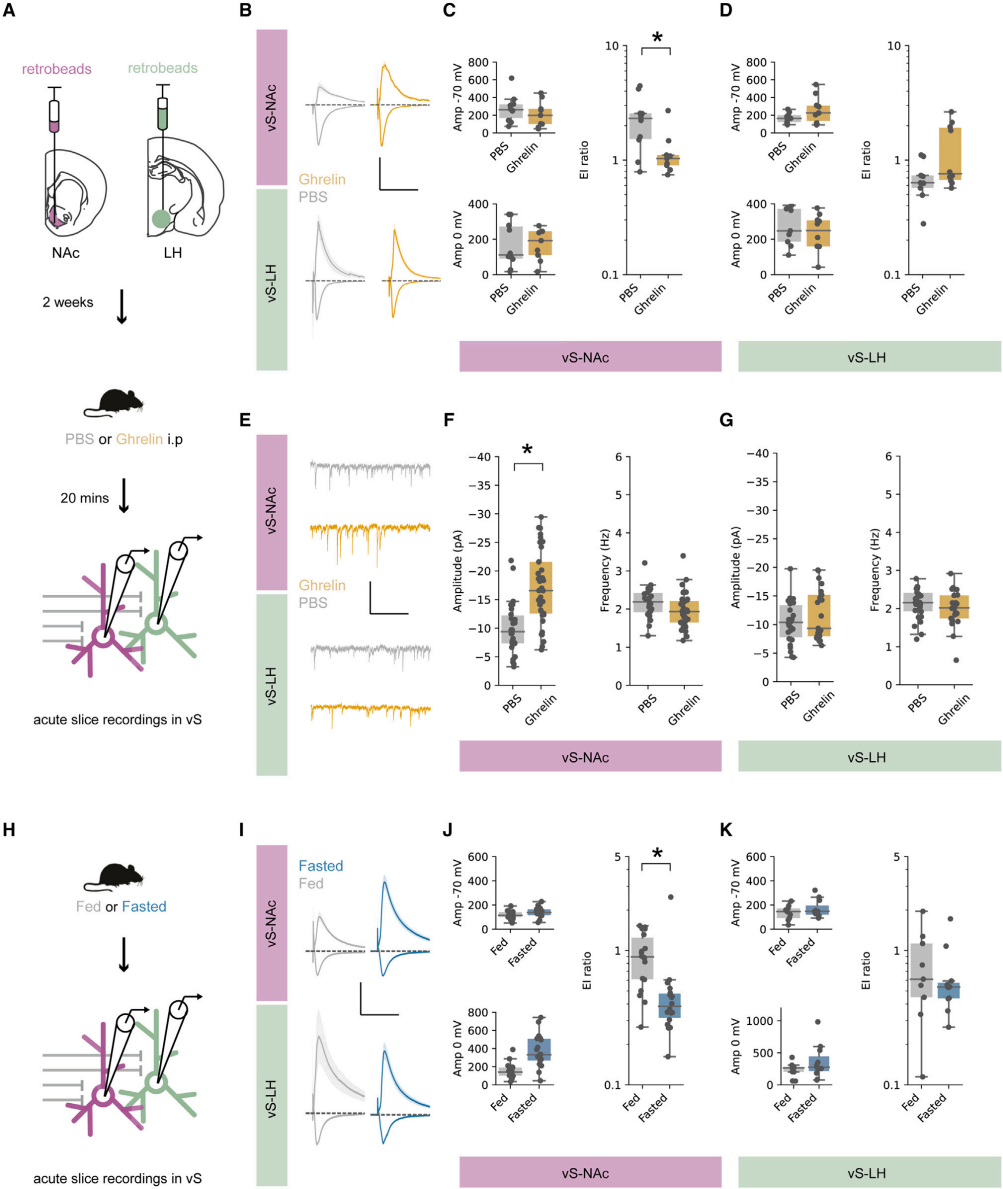
Ghrelin increases the amplitude of postsynaptic inhibition in vS-NAc neurons (A) Schematic of retrobead injections. (B) Average electrically evoked PSCs at −70 (downward trace) and 0 mV (upward trace) in vS-NAc and vS-LH neurons after PBS (gray) or ghrelin (gold) treatment. Scale bars, 200 pA, 100 ms. (C) Left: summary of PSC amplitude in vS-NAc at −70 (top) and 0 mV (bottom). Right: summary of E:I ratio (amplitude at −70 mV/amplitude at 0 mV). n = 10 neurons from 3 mice in PBS group, 9 neurons from 3 mice in ghrelin group. (D) As in (C) but for vS-LH neurons. n = 10 neurons from 3 mice in PBS group, 9 neurons from 3 mice in ghrelin group. (E) Example traces containing isolated mIPSCs in vS-NAc and vS-LH neurons after PBS (gray) or ghrelin (gold). Scale bars, 20 pA, 1 s. (F) Summary of amplitude (left) and frequency (right) of mIPSCs in vS-NAc neurons. n = 26 neurons from 3 mice in PBS group, 37 neurons from 5 mice in ghrelin group. (G) As in (F) but for vS-LH neurons. n = 26 neurons from 3 mice in PBS group, 21 neurons from 3 mice in ghrelin group. (H–K) As in (A)–(D), but showing responses at −70 and 0 mV for vS-NAc and vS-LH neurons in fed or fasted mice. For vS-NAc: n = 18 neurons from 3 mice in fed group, 18 neurons from 3 mice in fasted group; for vS-LH: n = 9 neurons from 3 mice in fed group, 11 neurons from 3 mice in fasted group. Scale bars, 200 pA, 100 ms. Boxplots represent the median, 75^th^, and 95^th^ percentiles, and individual datapoints are superimposed for clarity.

Our results so far suggest a model where a high level of eating after increases in peripheral ghrelin is associated with inhibition of vS-NAc activity during food investigation. We next wanted to look for the cellular underpinnings of this change. We hypothesized that this decrease in activity may be due to ghrelin-induced plasticity of inhibitory connectivity. To test this, we performed whole-cell recordings from fluorescently identified NAc- and LH-projecting vS neurons in acute slices (Figure 4A).

We first compared the relative excitatory and inhibitory synaptic strength on each projection population by calculating the E:I ratio in response to electrical stimulation of the Schaffer collateral input (the ratio of the mainly excitatory current at −70 mV divided by the mainly feedforward inhibitory current at 0 mV; Figure 4B). Interestingly, we found that while ghrelin administration had no influence on E:I ratio in vS-LH neurons, in vS-NAc neurons there was a large decrease in E:I ratio in mice treated with ghrelin compared with controls, suggesting an increase in relative inhibitory synaptic strength (Figures 4C and 4D).

Next, we recorded miniature inhibitory post synaptic currents (mIPSCs) from both vS-NAc and vS-LH neurons in control and ghrelin-treated mice (Figure 4E). In these recordings the amplitude of mIPSCs is proportional to the postsynaptic efficacy, while the frequency of events is proportional to both the probability of release and the number of synaptic connections. Consistent with our results above, we found that ghrelin resulted in a large increase in inhibition in vS-NAc neurons, but no changes in vS-LH neurons (Figures 4F and 4G). This increased inhibition was due to an increase in the amplitude of mIPSCs in vS-NAc neurons, with no change in their frequency (Figure 4F). Thus, ghrelin administration results in an increase in the postsynaptic efficacy of inhibition in vS-NAc neurons.

We next asked whether these changes in inhibitory synaptic strength were accompanied by changes in excitatory connections. We recorded spontaneous excitatory postsynaptic currents (sEPSCs) from both vS-NAc and vS-LH neurons (Figures S4A–S4C) and found no changes in either amplitude or frequency of these events in either population of neurons (Figures S4D and S4E).

Interestingly, in contrast to the administration of ghrelin *in vivo*, bath application of ghrelin to acute slices of the vS *in vitro* had little effect on inhibitory synaptic properties, or the intrinsic electrophysiological properties of either vS-NAc neurons or vS-LH neurons, at multiple concentrations (Figures S4F–S4M). Thus, changes in inhibitory synaptic connectivity in vS-NAc neurons require more than simply rises in ambient ghrelin concentration (see discussion).

Finally, our results so far have suggested that ghrelin administration results in very similar behavioral changes and alterations to vS activity to those induced by natural fasting. Therefore, we wanted to investigate whether similar changes in vS synaptic connectivity occurred in fasted compared with fed mice. To do this, we recorded from vS-NAc and vS-LH neurons in either fed and fasted mice, and again compared the E:I ratio in response to electrical stimulation of the Schaffer collateral input. Similar to mice with ghrelin administration, fasting had no influence on E:I ratio in vS-LH neurons but in vS-NAc neurons resulted in a large decrease in E:I ratio compared with fed controls, indicative of a similar increase in inhibitory synaptic strength (Figures 4H–4K).

Together, our experiments suggest that ghrelin administration, as well as natural fasting, increases synaptic inhibition onto vS-NAc neurons through an increase in the postsynaptic strength of inhibitory synapses.

### Artificially increasing vS-NAc activity blocks ghrelin-mediated increases in feeding

Our results so far suggest a model where increases in peripheral ghrelin are associated with inhibition of vS-NAc activity, and this inhibition results in increases in the transition from food investigation to food consumption. This is reminiscent of a much-hypothesized role for vS-NAc neurons in behavioral inhibition,^64^ where increased activity of vS-NAc neurons inhibits non-optimal ongoing behavior.^63^^,^^65^

**Figure 5:**
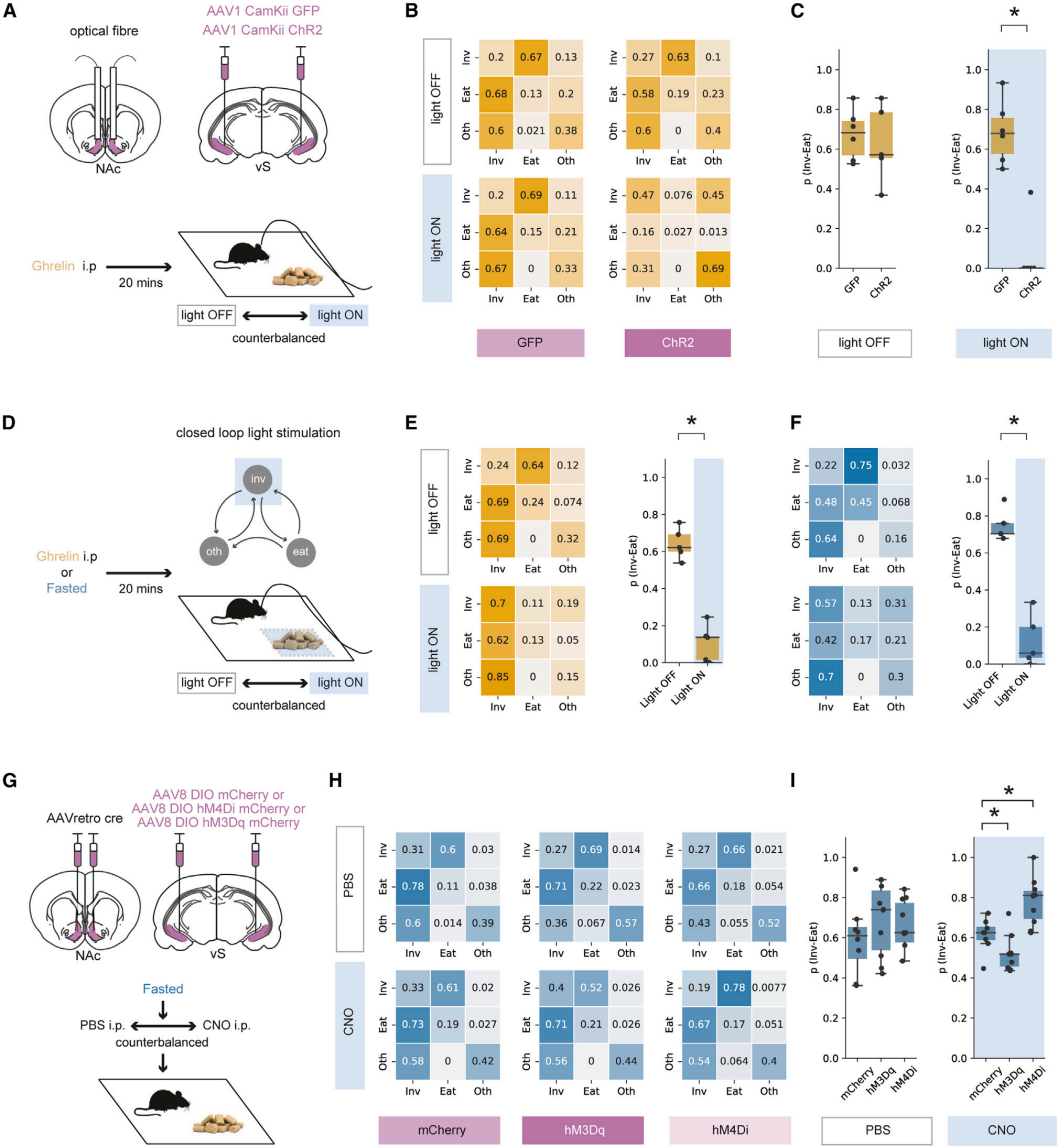
Activation of vS-NAc neurons blocks the transition from investigation to eating (A) Top: schematic of injections. Bottom: schematic of experiment. (B and C) Markov analysis of feeding behavior during 10-min session in GFP (n = 6) and ChR2 (n = 5) mice, with or without light stimulation. Left: state transitions for light OFF and light ON sessions. Right: summary of investigation to eat transition across all mice, with and without light. (D) Schematic of experiment. (E) Markov analysis of feeding behavior during 10-min session after ghrelin (n = 5). Left: state transitions for light OFF and light ON sessions. Right: summary of investigation to eat transition across all mice, with and without light. (F) As in (E) but for overnight-fasted mice (n = 5). (G) Top: schematic of injections. Bottom: schematic of experiment. (H and I) Markov analysis of feeding behavior during 10-min session in mCherry (n = 8), excitatory hM3D (n = 10), and inhibitory hM4D (n = 10) expressing mice after PBS (top) or CNO (bottom). (H) State transitions for PBS and CNO sessions. (I) Summary of investigation to eat transition across all mice with either PBS or CNO injections. Boxplots represent the median, 75^th^, and 95^th^ percentiles, and individual datapoints are superimposed for clarity.

We reasoned that, if activity of vS-NAc neurons specifically inhibited the transition from investigation to eating, artificial activation of vS-NAc neurons should block ghrelin-induced increases in feeding but have little effect on other behaviors—in particular the frequency of investigation of food. To test this, we expressed the excitatory opsin channelrhodopsin2 (ChR2) bilaterally in excitatory neurons in the vS, allowing us to bilaterally activate vS terminals in the NAc with brief pulses of blue light (Figures 5A, S5A, and S5B). We first compared mice expressing ChR2 with control mice expressing GFP. We repeated the 10-min feeding assay in a counterbalanced design, where in both cases the mouse was given ghrelin administration. We then scored behavior as before across each session, but on 1 day the mouse underwent constant 20-Hz blue light stimulation during the session, while on the alternate day no light was present (Figure 5A).

In GFP control animals, there was no effect of blue light stimulation, and ghrelin resulted in robust feeding behavior in both light ON and light OFF days (as seen by measures such as high chow consumption and high frequency of investigation of food; Figures S5C–S5E). Similarly, by creating transition matrices for each animal in each condition, we found that there was a high probability of transitioning from investigation to eating p_(Inv→Eat)_ and that this was unaltered by light stimulation (Figures 5B and 5C). However, light delivery in ChR2-expressing animals caused large but specific changes in behavior. Although light stimulation had little influence on the frequency of food investigation or locomotor behaviors such as velocity of movement (Figures S5C–S5E), it resulted in an almost complete cessation of eating (Figures S5C and S5D). Again, by constructing transition matrices for these animals we found that this was due to a marked reduction in p_(Inv→Eat)_ (Figures 5B and 5C). Together, these results suggest that activation of vS-NAc neurons blocks the transition from food investigation to eating, even in the presence of high levels of peripheral ghrelin.

We next took advantage of the temporal specificity of the optogenetic stimulus and used a closed-loop paradigm to investigate the influence of optogenetic activation of vS-NAc terminals only when the mouse was investigating food—mimicking the transient increases in vS-NAc activity seen in our photometry experiments (Figure 5D). Using this paradigm, we again found that activating vS-NAc neurons only during investigation markedly lowered p_(Inv→Eat)_ (Figure 5E) compared with control OFF days with no stimulation and that this effect was similar at both the start and the end of the 10-min session (Figures S5G–S5I). On stimulation ON days, we also monitored behavior for 5 min after we had ceased light stimulation and found that mouse behavior returned to baseline almost instantly (Figures S5G–S5I), suggesting that while vS-NAc activity is very effective at reducing p_(Inv→Eat)_, this activity needs to be maintained in order to influence behavior. Finally, we noted that in this closed-loop paradigm, mice also increased the frequency of food investigation throughout the session (Figure S5F), resulting in the majority of the session spent investigating food, albeit with a very low likelihood of each investigation transitioning to eating (Figure 5E).

Our results so far suggest that artificial increases in vS-NAc activity can block ghrelin-induced increases in feeding behavior. Our previous behavioral, imaging, and electrophysiology data suggest that similar mechanisms may be involved in controlling increased feeding behavior due to natural hunger. Therefore, we repeated our closed-loop experiment in overnight-fasted mice (Figure 5F) and showed that, consistent with our experiments utilizing ghrelin administration, increasing vS-NAc activity around the investigation of food in fasted mice markedly decreased p_(Inv→Eat)_ (Figures 5F and S5F), and this effect immediately returned to baseline upon cessation of the optogenetic stimulus (Figure S5J).

Our optogenetic stimulation experiments show that large, artificial changes in vS-NAc activity can inhibit ghrelin- and fasting-induced increases in p_(Inv→Eat)_. We next wanted to investigate whether more subtle manipulations could also influence behavior in a similar way. To achieve this, we took advantage of the designer receptors exclusively activated by designer drugs (*DREADDs*), hM3D and hM4D, which upon activation engage excitatory and inhibitory second messenger cascades, respectively,^72^ allowing us to induce a more physiological, bidirectional modulation of vS-NAc activity. We utilized intersectional approaches to express either hM3D-mCherry, hM4D-mCherry, or mCherry alone as a control only in vS-NAc neurons (Figures 5G, S5K, and S5L), and compared behavior in fasted mice after a counterbalanced intraperitoneal (i.p.) injection of the DREADD agonist clozapine-N-oxide (CNO) or control PBS. Although we found no changes to behavior in PBS-treated mice (Figure S5), in CNO-treated mice we saw a bidirectional change in p_(Inv→Eat)_ that was consistent with our optogenetic findings, where activation of vS-NAc neurons with hM3D decreased p_(Inv→Eat)_ compared with mCherry controls and decreasing activity with hM4D increased p_(Inv→Eat)_. These changes occurred with minimal effects on other behaviors (Figures 5H and S5O–S5Q) and with no change in overall locomotor activity (Figure S5R).

Finally, we explicitly investigated how activation or inactivation of these neurons influenced total food intake in fasted mice, using a home cage feeding assay (Figure S5S). We measured total chow consumption regularly over the course of 24 h in hM3D-, hM4D-, or mCherry-expressing mice treated with CNO. Interestingly, there was no effect of altering vS-NAc activity on food consumption (Figures S5T–S5V). Thus, vS-NAc activity can bidirectionally define the probability of transitioning from food investigation to consumption but has minimal influence on overall levels of consumption over long timescales (see discussion).

Together, our activity manipulations suggest that a key effect of peripheral ghrelin in the vS is to inhibit the activity of vS-NAc neurons, which allows the animal to overcome a “block” that this activity imposes on the initiation of feeding behavior.

### GHSR1a expression in vS-NAc neurons is required for ghrelin-mediated changes in inhibitory synaptic strength

We next wanted to understand the mechanism by which peripheral ghrelin can influence synaptic inhibition in vS-NAc neurons. vS neurons express the ghrelin receptor GHSR1a, and peripheral ghrelin is known to cross the BBB and enter the hippocampus, where it has the ability to directly influence postsynaptic properties to influence behavior.^47^^,^^48^^,^^54^^,^^55^^,^^56^ Therefore, we wanted to ask whether the influence of peripheral ghrelin we observed on vS inhibitory synaptic properties might be via this direct activation of the GHSR1a receptor on the postsynaptic membrane of vS-NAc neurons.

**Figure 6:**
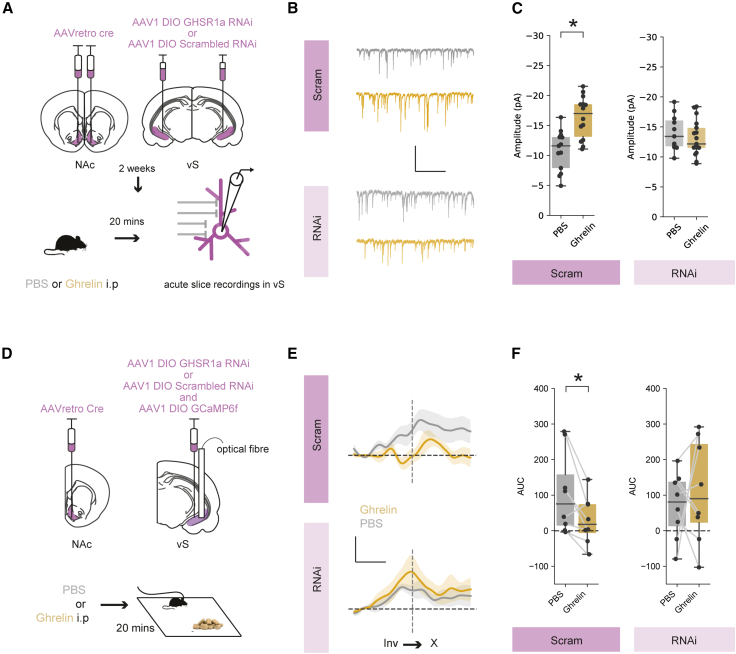
Postsynaptic expression of the ghrelin receptor GHSR1a is required for inhibition of vS-NAc neurons (A) Top: schematic of injections. Bottom: schematic of experiment. (B) Example traces containing isolated mIPSCs in vS-NAc neurons expressing GHSR1a RNAi or scrambled control after PBS (gray) or ghrelin (gold). Scale bars, 15 pA, 1 s. (C) Summary of amplitude of mIPSCs in vS-NAc neurons. For scrambled: n = 13 neurons from 3 mice in PBS group, n = 14 neurons from 3 mice in ghrelin group; for RNAi: n = 11 neurons from 3 mice in PBS group, n = 17 neurons from 3 mice in ghrelin group. (D) Top: schematic of injections. Bottom: schematic of experiment. (E) Average activity of vS-NAc neurons across all mice expressing GHSR1a RNAi (n = 8) or scrambled control (n = 8), aligned to end of food investigation after injection of either PBS (gray) or ghrelin (gold). Scale bars, 0.5 zF, 4 s. (F) Summary of activity around investigation for vS-NAc neurons expressing GHSR1a RNAi or scrambled control after PBS (gray) or ghrelin (gold). Boxplots represent the median, 75^th^, and 95^th^ percentiles, and individual datapoints are superimposed for clarity.

To test this, we developed a viral vector containing a cre-dependent RNA interference (RNAi) cassette that contained an shRNAmiR sequence that knocked down expression of GHSR1a (Figures S6A and S6B), along with a fluorescent reporter for visualization. We then used an intersectional viral method as before to reduce levels of GHSR1a only in vS-NAc neurons (Figures 6A and S6C–S6F), and used an RNAi targeted to a scrambled sequence as a control. Thus, in these animals, GHSR1a is knocked down only in vS neurons that project to the NAc, allowing us to investigate the influence of GHSR1a in these neurons, while leaving GHSR1a expression in other canonical regions, such as the arcuate nucleus and LH, intact. After allowing for expression, we prepared acute slices from these animals after i.p. injection with either PBS or ghrelin as before. We then recorded mIPSCs from identified vS-NAc neurons expressing the GHSR1a or control RNAi constructs, identified by mCherry fluorescence.

Consistent with our previous results, we found that in mice expressing the scrambled control RNAi in vS-NAc neurons, ghrelin administration resulted in an increase in mIPSC amplitude (Figures 6B and 6C). However, in neurons with knockdown of GHSR1a, mIPSCs were almost completely insensitive to ghrelin administration (Figures 6B and 6C). Therefore, the changes in inhibitory synaptic connectivity in vS-NAc neurons on administration of peripheral ghrelin require expression of the GHSR1a receptor on the postsynaptic membrane of vS-NAc neurons.

### vS-NAc GHSR1a expression is required for peripheral ghrelin-induced alterations in vS-NAc activity during feeding

Our results above suggested that increased synaptic inhibition in vS-NAc neurons after ghrelin treatment is mediated via postsynaptic GHSR1a. Our model proposes that the reduction of vS-NAc activity during investigation of food induced by ghrelin is due to this increased inhibitory drive. If this were true, then GHSR1a knockdown should also block this ghrelin-mediated reduction in activity. To test this, we again used intersectional viral infection to unilaterally express either control or GHSR1a RNAi constructs in vS-NAc neurons (Figure 6D). In each mouse, we also expressed GCaMP6f in overlapping vS-NAc neurons (∼80% of neurons were co-labeled with both the RNAi construct and GCAMP6f; Figures S6C–S6F). Importantly, due to redundancy across hemispheres, unilateral expression of the RNAi constructs ensured that behavior was not affected by the manipulation of GHSR1a levels (Figures S6G and S6H) and so allowed investigation of alterations in vS-NAc activity under equivalent behavioral conditions. Together, this approach allowed us to use fiber photometry to monitor the activity of vS-NAc neurons with or without GHSR1a knockdown during feeding behavior and to investigate the influence of peripheral ghrelin administration on this activity.

We first found that sated mice expressing the control RNAi again showed a ramp-up in activity around investigation and that this was reduced after ghrelin administration (Figures 6E and 6F). In contrast, the activity of vS-NAc neurons in GHSR1a knockdown animals during food investigation remained high even after ghrelin administration (Figures 6E and 6F). Together, this suggests that GHSR1a in vS-NAc neurons is required for peripheral ghrelin to increase inhibitory synaptic strength in vS-NAc neurons and that removal of GHSR1a renders vS-NAc activity during investigation of food insensitive to peripheral ghrelin.

### vS-NAc GHSR1a is required for peripheral ghrelin-mediated increase in the transition from investigation to eating

Our results suggest that GHSR1a is required for peripheral ghrelin to inhibit the activity of vS-NAc neurons around food investigation. Moreover, our activity manipulation experiments (Figure 5) show that this reduction in vS-NAc activity is necessary for ghrelin-mediated increases in transitioning from food investigation to food consumption. Therefore, we hypothesized that GHSR1a in vS-NAc neurons would be a key factor in ghrelin-mediated changes in feeding behavior.

**Figure 7:**
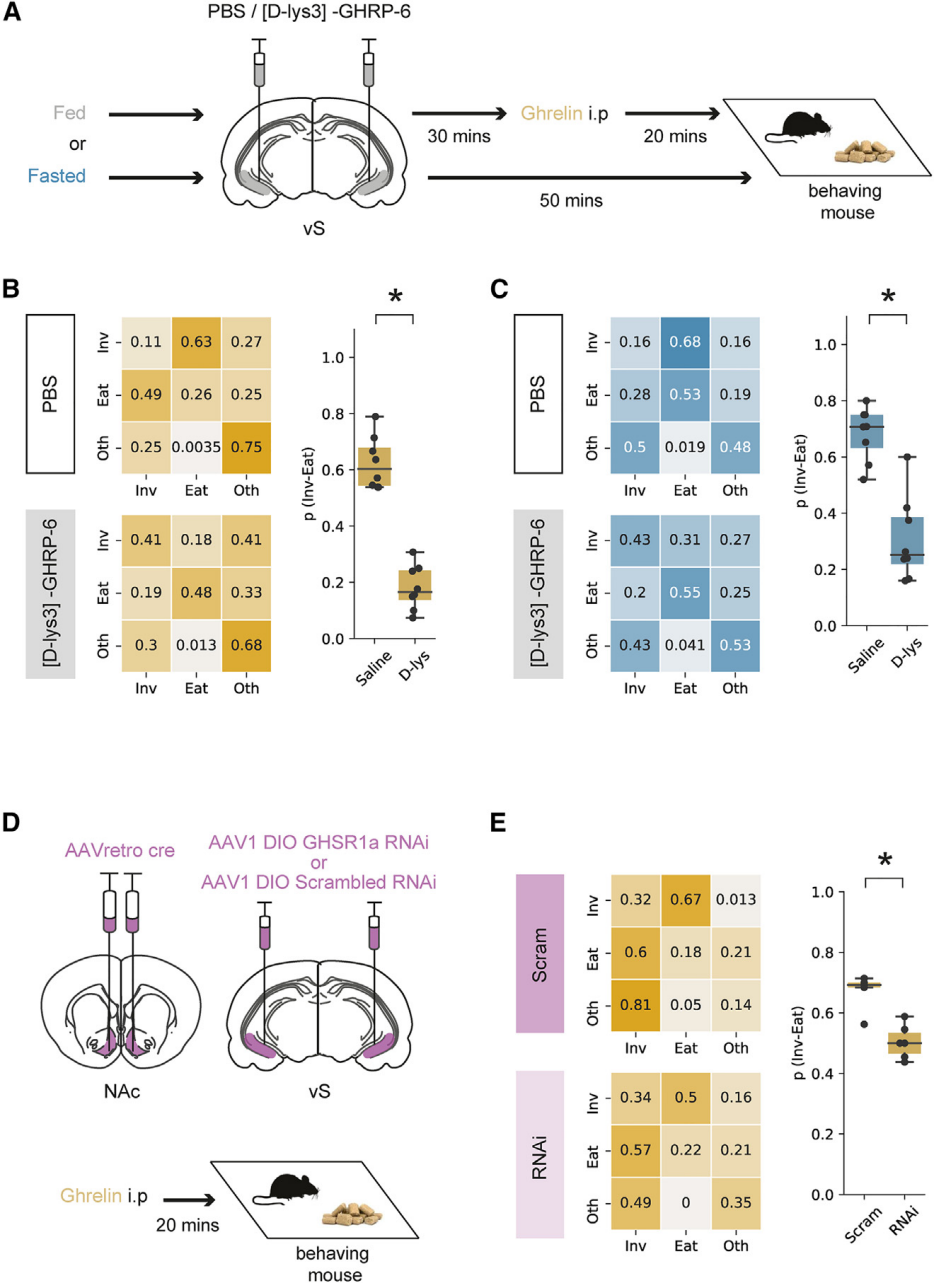
Postsynaptic GHSR1a expression in vS-NAc neurons is required for ghrelin-induced increase in the transition from food investigation to eating (A) Schematic of experiment. (B) Markov analysis of feeding behavior in ghrelin-treated mice during 10-min session after injection of either PBS (n = 8) or D-Lys (n = 8) in the vS. Left: state transitions for PBS (top) and D-Lys (bottom). Right: summary of investigation to eat transition across all mice. (C) As in (B) but for overnight-fasted mice. (D) Left: schematic of injections. Right: schematic of experiment. (E) Markov analysis of feeding behavior during 10-min session in ghrelin-treated mice with either GHSR1a RNAi (n = 6) or control (n = 6). Left: state transitions for control (top) and RNAi (bottom). Right: summary of investigation to eat transition across all mice. Boxplots represent the median, 75^th^, and 95^th^ percentiles, and individual datapoints are superimposed for clarity.

To test this, we first compared the behavior of mice after ghrelin administration, with or without vS GHSR1a function. To do this, we used implanted cannulae to inject either the ghrelin antagonist [D-lys3]-GHRP-6, or control PBS, counterbalanced across days, in the vS before ghrelin administration (Figures 7A and S7A). Consistent with a key role for vS GHSR1a, antagonist injection resulted in a large reduction in p_(Inv→Eat)_ compared with PBS control (Figure 7B), with minimal effect on other behaviors (Figure S7C)—similar to the effect observed in our vS activity manipulations. Moreover, consistent with a more general role in control of hunger-induced behavior, we observed a similar effect of vS GHSR1a antagonism in naturally fasted mice (Figures 7C and S7D). Together, this suggests that a key means of controlling anticipatory behavior around food is GHSR1a signaling in the vS.

Our previous results suggested that the effect of ghrelin in the vS was specifically via the action of vS-NAc neurons. To investigate the role of GHSR1a function, specifically in this projection population, we intersectionally expressed GHSR1a RNAi bilaterally in vS-NAc neurons and compared this to littermate controls with control RNAi expression (Figures 7D, S7E, and S7F). In comparison with our previous unilateral knockdown experiments designed to not affect behavior, the bilateral expression in this experiment does not allow for compensation by the non-manipulated hemisphere and can therefore be used to probe behavioral function (in contrast to Figures S6G and S6H). We then used this approach to investigate the influence of GHSR1a knockdown on feeding in response to peripheral ghrelin.

We first looked at the effect of GHSR1a knockdown on the frequency of feeding-associated behaviors. We found no effect of GHSR1a knockdown on either investigation of food or feeding in PBS-treated mice (Figures S7G and S7H). This suggests that there is little role for constitutive GHSR1a activity in sated mice, potentially due to a strong floor effect.^73^ However, in ghrelin-treated animals, while both control and GHSR1a RNAi animals showed a similar frequency of investigation of food, GHSR1a RNAi expression was accompanied by a reduction in the frequency of eating compared with control animals (Figure S7J)—similar to the effect of artificially activating vS-NAc neurons (Figure 5). Similarly, by constructing transition matrices for each animal in each condition, we again saw that vS-NAc GHSR1a knockdown resulted in a consistent and specific reduction of p_(Inv→Eat)_ (Figures 7D and 7E).

Overall, we have shown that peripheral ghrelin increases inhibitory synaptic strength on vS-NAc neurons via a mechanism that requires GHSR1a. This increased inhibition accompanies reduced vS-NAc activity during investigation of food, and this reduction of activity promotes the transition from investigation to consumption of food.

## Discussion

### The role of ghrelin in anticipatory versus consummatory behavior

Our data demonstrate that ghrelin inhibits vS activity specifically during food anticipation and suggest that it is this inhibition that promotes the transition to eating. On the surface, this goes against classic notions of ghrelin as a “hunger hormone” that mimics fasting by increasing food consumption.^74^^,^^75^ However, this is consistent with several observations suggesting that ghrelin mediates food anticipation rather than food consumption. For example, the concentration of circulating ghrelin accurately reflects the anticipation of an upcoming meal,^76^^,^^77^ and studies employing pharmacological and genetic disruption of GHSR1a, both systemically and in the vS, show that mice have disrupted anticipatory behavior preceding a meal.^55^^,^^78^^,^^79^ Furthermore, GHSR1a-null mice have normal body weight,^80^ indicating that ghrelin is dispensable in homeostatic food intake regulation. Consistent with these findings, we found that manipulating vS-NAc activity had no long-term influence on overall consumption of food (Figure S5), reinforcing the idea that the influence of ghrelin in the vS is in the anticipation of food, not its consumption. However, in our simple experimental setup, it was not possible to investigate the interaction between these two stages of feeding behavior definitively. For example, in the short sessions we utilized in our study, changes in p_(Inv→Eat)_ affect total chow consumption simply due to there being fewer occasions with the possibility to consume food. As a result, in many of our experimental manipulations, we found both changes in p_(Inv→Eat)_ as well as changes in total chow consumption. Therefore, future work with more sensitive measures of feeding behavior allowing more nuanced separation of factors such as meal size, motivation to feed, and satiety—and a potential role of different parts of vS circuitry in these factors—will be extremely interesting.^9^^,^^10^

### Mechanisms of ghrelin signaling in the vS

Given the tight regulation of substance entry across the BBB, one important question is whether ghrelin and other hormones mediate their effects on vS circuitry by directly binding hippocampal neurons or are instead signaled indirectly via upstream synaptic inputs that themselves have access to peripheral ghrelin. The hippocampus is situated adjacent to circulating cerebrospinal fluid (CSF) in the ventricles and has a rich surrounding blood supply from the choroid plexus^44^ that facilitates the transfer of molecules across the BBB,^51^ including ghrelin.^48^^,^^52^ This is consistent with not only a vast array of peripheral ghrelin-mediated structural and functional effects on hippocampal neurons^48^^,^^54^ but also the role of hippocampal ghrelin in influencing behavior.^47^^,^^48^^,^^55^ More generally, the hippocampus expresses functional receptors for a multitude of peptide hormones similar to ghrelin,^44^ such as leptin^81^ and insulin.^82^ Together, this suggests that these hormones are likely capable of binding to hippocampal neurons to affect their function.

It is important to note, however, that the influence of ghrelin on vS circuitry may occur via other, indirect mechanisms. First, there is a line of evidence that proposes that peptide hormones like ghrelin, insulin, and leptin are unable to cross the BBB^53^^,^^73^ without specialized mechanisms,^52^ and it is unclear whether these are present in the vS. Although the contradictory findings surrounding ghrelin access to the vS could be explained by BBB permeability being extremely plastic (for example, accessibility of ghrelin is itself often dependent on hunger state^83^^,^^84^), there remains a possibility that direct permeability through the BBB might not be the major route for ghrelin to influence the vS.

As a result, multiple alternate mechanisms have been proposed to explain the role of GHSR1a signaling in the vS. First, it has been proposed that the ghrelin receptor GHSR1a exerts its effect through ligand-independent constitutive activity.^85^ However, this is hard to reconcile with past experiments showing the presence of peripheral ghrelin in the hippocampus,^48^ as well as other experiments,^47^^,^^55^ including ours (Figures 6 and 7), showing an active role of the receptor in response to stimuli.

Interestingly, while ghrelin administration *in vivo* altered vS-NAc inhibitory synaptic properties (Figure 4), bath application of ghrelin to acute slices *in vitro* did not recapitulate this effect (Figure S4). This may be due to multiple factors. First it may be that ghrelin mediates its effect solely through downstream input and not through direct influence on the vS, although, as described above, this appears unlikely to be wholly the case. Second, similar to plasticity of excitatory synapses, inhibitory plasticity depends on coincidence between highly controlled changes in postsynaptic signaling and tightly controlled trains of presynaptic activity^86^; thus, this lack of an *in vitro* effect may be due to the requirement for precise pre- and postsynaptic activity to accompany changes in vS ghrelin tone in order to induce plasticity. Finally, an alternative explanation may arise through the proposed dimerization of the GHSR1a receptor with the D1 dopamine receptor,^73^ which could render vS ghrelin signaling co-dependent on dopamine and, conversely, vS dopamine signaling dependent upon GHSR1a expression. In this scenario, as ghrelin also indirectly activates ghrelin-sensing LH neurons that promote activity in the ventral tegmental area,^87^^,^^88^ this activity is well placed to provide a coincident dopaminergic input to the hippocampus. This requirement for the combinatorial presence of coincident signaling systems is common across the brain.^89^^,^^90^^,^^91^ As the vS receives a large amount of neuromodulatory input,^90^ other neuromodulators or neuropeptides could also act as co-agonists to ghrelin-mediated plasticity in the vS, including acetyl-choline from septal areas, serotonin from raphe, or melanin-concentrating hormone from the hypothalamus.^92^^,^^93^^,^^94^ Future studies investigating the exact requirements for ghrelin-induced inhibitory plasticity are needed to resolve this interesting question.

### The role of the vS in hunger-sensitive goal-directed behavior

It is becoming increasingly understood that the hippocampus not only encodes the relationships between distinct cues in the environment^18^^,^^95^ but also the value of outcomes^96^^,^^97^—and their interaction. For example, the hippocampal-to-NAc projection has been proposed to be important for the learning of value associations in both physical and abstract dimensions^32^^,^^33^^,^^58^^,^^98^^,^^99^ and to relay these signals to ventral striatum.^33^^,^^58^^,^^100^^,^^101^ This ability is proposed to allow the utilization of past experience to anticipate the outcome of upcoming behavior. Consistent with this proposed role, we found that vS-NAc was active around the investigation of salient objects and that this activity ramps up during investigative behavior, consistent with studies of spatial goal-directed navigation.^34^ This ramping activity was observed during investigations of non-food objects and across food of different palatabilities, consistent with a more general role of vS-NAc activity in goal-directed behaviors, including social interaction^102^ and reward-place associations.^32^^,^^33^^,^^58^

When investigating food, vS-NAc activity around investigation was anticorrelated with the overall amount of food consumed during the session^34^^,^^63^^,^^93^^,^^103^ (Figure 3). Interestingly, while this correlation was present on a session-by-session timescale, it was not apparent on a moment-to-moment timescale—vS-NAc activity was not different over individual investigations that resulted in consumption and those that did not (Figure S3). This long-term signaling of internal state is consistent with the idea of vS providing a stable context upon which to base the learned consequences of cues and actions that are conditionally ambiguous.^13^^,^^17^

Interestingly, this reduction in anticipatory vS-NAc activity during investigation seemed to be specific to the investigation of food, as activity was unaltered around investigation of non-food objects, activity upon food consumption, and activity around other non-feeding-related behaviors such as rearing (Figures 3 and S3). Therefore, while vS-NAc activity is responsive to multiple features, such as investigation of non-food objects and the initiation of eating,^63^^,^^102^ and manipulation of vS activity is sufficient to directly influence consummatory behavior,^63^^,^^93^ ghrelin signaling in the vS influences only the anticipatory activity leading up to food consumption. Future work is needed to investigate the mechanisms underlying this specificity.

In contrast to the modulation of vS-NAc activity and synaptic properties by ghrelin, we found that such changes were not present in neighboring vS-LH neurons (Figures 2, 3, and 4), which seemed instead to be activated by salient events such as presentation of food or non-food items (Figure S2). This observation fits well with increasing evidence showing a role for LH in salience detection^69^ as well as studies showing that activation of this pathway increases acute anxiety-like behaviors and place aversion,^25^ often associated with novel, salient situations. Interestingly, consistent with a lack of activity around chow in our study, projections from the vS to other hypothalamic regions are also not responsive around consumption of regular chow and are instead involved in the learned associations of highly palatable food.^14^ As such, in the future it will be interesting to investigate the unique role of this projection and how the potential representations of salience, threat, and palatability in vS-LH neurons compliment and contrast with the role for vS-NAc neurons we have identified in this study.

Finally, downstream of the vS, our work aligns well with a proposed circuit mechanism for the rapid control of feeding behavior promoted by activity of D1 dopamine-receptor-expressing medium spiny neurons (D1 MSNs) in the NAc.^63^^,^^65^^,^^103^ vS input to the NAc preferentially targets D1 MSNs,^101^^,^^104^ activity of which can rapidly suppress reward consumption and promote exploratory and seeking behaviors.^31^ In particular, during consummatory behavior, a subpopulation of D1 MSNs that project to LH directly inhibit eating via the targeting of LH GABAergic neurons.^47^^,^^65^ Thus, through this specialized connectivity, vS-NAc ghrelin sensitivity is ideally situated to provide strong and acute control over feeding behavior and provides a means for this control to be utilized to shape behavior based on internal state.

### Internal state as a distinct dimension of the hippocampal representation

The hippocampus has long been viewed as circuit by which otherwise ambiguous cues can be separated through their association with other cues that occur in close spatial and temporal proximity.^19^ Although such representations are often studied in terms of sensory perceptions such as vision, audition, and olfaction, our work complements a number of studies suggesting that the internal state may also be used to disambiguate such situations.^13^^,^^14^^,^^15^^,^^16^^,^^36^^,^^37^^,^^38^^,^^39^^,^^40^ In this study, we show that ghrelin sensing in the vS has a key role in modulating behavior toward food, dependent on the internal state. However, it is likely that ghrelin sensing in the hippocampus is integrated with other modalities in order to be utilized more generally—for example, to allow the balance of approach and avoidance behavior, passive and active strategies in potentially threatening or novel situations,^59^^,^^105^ and context-dependent associative learning more generally.^14^^,^^15^^,^^16^^,^^106^^,^^107^

## STAR★Methods

### Key resources table

**Table:** 

REAGENT or RESOURCE	SOURCE	IDENTIFIER
**Bacterial and Virus Strains**		

pAAV2-retro-CAG-Cre	Tervo et al.^109^; UNC Vector Core	N/A
pENN-AAV-hSyn-Cre-WPRE-hGH	a gift from James M. Wilson	RRID:Addgene_105553
AAV1-CAG-Flex-GCaMP6f-WPRE-SV40	a gift from Douglas Kim & GENIE Project	RRID:Addgene_100833
pAAV-hSyn-hChR2(H134R)-EYFP	a gift from Karl Deisseroth	RRID:Addgene_26973
AAV1-CamKII-EYFP	a gift from Karl Deisseroth	RRID:Addgene_105622
AAV1-EF1a-DIO-mCherry-scrmb-shRNAmir	VectorBiolabs	Lot #191007#34
AAV1-EF1a-DIO-mCherry-ghsr-shRNAmir	VectorBiolabs	VB190712-1054mfw
AAV8-EF1a-DIO-mCherry	UNC Vector Core	N/A
AAV8-EF1a-DIO-mCherry-hM3Dq	UNC Vector Core	N/A
AAV8-EF1a-DIO-mCherry-hM4Di	UNC Vector Core	N/A

**Chemicals, Peptides, and Recombinant Proteins**		

Red retrobeads	Lumafluor	Item #: R180
Green retrobeads	Lumafluor	Item #: G180
Cholera Toxin Subunit B (Recombinant), Alexa Fluor 647 Conjugate	Invitrogen	Cat #C34778
Cholera Toxin Subunit B (Recombinant), Alexa Fluor 555 Conjugate	Invitrogen	Cat #C34776
ProLong Gold Antifade Mountant with DAPI	Invitrogen	Cat #P36930
ProLong Glass Antifade Mountant with DAPI	Invitrogen	Cat #P36984
Isoflurane	Piramal Critical Care	N/A
Carprofen	Norbrook	N/A
Tetrodotoxin	Hello Bio	Cat #HB1035

**Deposited Data**		

Processed data	This paper	https://github.com/MacAskillLab/Wee_2023_Hunger

**Experimental Models: Organisms/Strains**		

Mouse: C57BL/6	Charles River	N/A

**Software and Algorithms**		

ImageJ (Fiji) Software	https://fiji.sc/	N/A
Python 3.6	https://www.python.org/	N/A
Jupyter Notebook	https://jupyter.org/	N/A

### Resource availability

#### Lead contact

Further information and requests for resources and reagents should be directed to and will be fulfilled by the lead contact, Andrew MacAskill (a.macaskill@ucl.ac.uk).

#### Materials availability

This study did not generate new unique reagents.

#### Data and code availability

The processed data reported in this paper has been deposited online at https://github.com/MacAskillLab/Wee_2023_Hunger. Raw data will be shared by the lead contact upon reasonable request.

This study did not generate new original code. Photometry code previously reported^59^ has been deposited at https://github.com/MacAskillLab.

### Experimental model and subject details

#### Mice

Young adult C57BL/6J male mice (behavioural and anatomical experiments: at least 7 weeks old; whole-cell electrophysiology experiments: 7 – 9 weeks old) provided by Charles River were used for all experiments. Only male mice were used to control for the potential influence and interaction of other circulating hormones in vS such as oestrogen.^108^ Future work will be aimed at investigating these interactions and if the influence of ghrelin signalling in vS is consistent across sex. All animals were housed in cages of 2 to 4 in a temperature- and humidity-controlled environment with a 12 h light- dark cycle (lights on at 7 am to 7 pm). Food and water were provided ad libitum. All experiments were approved by the UK Home Office as defined by the Animals (Scientific Procedures) Act, and strictly followed University College London ethical guidelines.

### Method details

#### Viruses

**Table:** 

Construct	Titre
rAAV2-retro-Syn-Cre	5.3 × 1012
AAV1-CAG-Flex-GCaMP6f-WPRE-SV40	>1 × 1013
AAV1-CamKII-EYFP	>1 × 1013
pAAV-CamKII-hChR2(H134R)-EYFP	2.5 × 1013
AAV1-EF1a-DIO-mCherry-scrmb-shRNAmir	1.0 × 1013
AAV1-EF1a-DIO-mCherry-ghsr-shRNAmir	4.1 × 1013
AAV8-EF1a-DIO-mCherry	>1 × 1013
AAV8-EF1a-DIO-mCherry-hM3Dq	>1 × 1013
AAV8-EF1a-DIO-mCherry-hM4Di	>1 × 1013

#### Stereotaxic surgery

Stereotaxic surgeries were performed according to previously described protocols.^57^^,^^58^^,^^59^ Mice were anaesthetised with isoflurane (4% induction, 1.5 to 2% maintenance) and secured onto a stereotaxic apparatus (Kopf). A single incision was made along the midline to reveal the skull. AP, ML and DV were measured relative to bregma, and craniotomies were drilled over the injection sites.

Stereotaxic coordinates:

**Table:** 

Region	ML	AP	DV
Lateral hypothalamus	0.9	-1.3	-5.2
Nucleus accumbens (medial shell)	0.9	+1.1	-4.6
Ventral subiculum	3.4	-3.2	-4.3

Long-shaft borosilicate pipettes were pulled and backfilled with mineral oil, and viruses were loaded into the pipettes. Viruses were injected with a Nanoject II (Drummond Scientific) at a rate of 13.8 to 27.6 nL every 10 s. Following infusion of the virus, the pipette was left in place for an additional 10 mins before being slowly retracted. For anatomy experiments, following injection of substances into the brain, animals were sutured and recovered for 30 mins on a heat pad. Animals received carprofen as a peri-operative s.c. injection (0.5 mg/kg) and in their drinking water (0.05 mg/mL) for 48 hours post-surgery.

For photometry, optogenetic and cannula-based injection experiments, cannulae were implanted following virus injection in the same surgery. The skull was roughened and two metal screws were inserted into the skull to aid cement attachment. Photometry cannulas were targeted to ventral CA1/subiculum, optogenetic cannulas were inserted at a 10-degree angle targeted to NAc shell. Cannulas were secured to the skull by applying two layers of adhesive dental cement (Superbond CB). The skin was attached to the cured dental cement with Vetbond. Animals received a subcutaneous injection of carprofen (∼5 μL of 0.5 mg/mL stock) prior to recovery in a warm chamber for 1 hour and continued receiving carprofen in their drinking water (0.05 mg/mL) for 48 hours post-surgery. Mice were allowed to recover for a minimum of 3 weeks before starting photometry experiments. For projection-specific expression of GCaMP6f, 150 – 200 nL of rAAV2-retro-Syn-Cre^109^ was injected into the output site (LH or NAc); in the same surgery, 300 – 400 nL of a 1:3 dilution of AAV1-CAG-Flex-GCaMP6f-WPRE-SV40 in sterile saline was injected into vS. This dilution protocol was used to delay excessive GCaMP expression, which could lead to reduced Ca2+ variance in the signal, affect cellular processes and reduce cell health.^110^ For optogenetic experiments, 400 nL of either AAV1-CamKII-hChR2(H134R)-EYFP, or AAV1-CamKII-GFP as a control were injected into vS. For combined projection-specific fibre photometry and molecular knockdown experiments, 150 – 200 nL rAAV2-retro-Syn-Cre was injected into NAc, and a 1:1 mix (400 nL) of AAV1-CAG-Flex-GCaMP6f-WPRE-SV40 and AAV1-EF1a- DIO-mCherry-ghsr-shRNAmir or AAV1-EF1a-DIO-mCherry-scrmb-shRNAmir was injected into vS. For bilateral knockdown of GHSR1a, 200 nL rAAV2-retro-Syn-Cre was injected into NAc, and 800 nL of AAV1-EF1a- DIO-mCherry-ghsr-shRNAmir or AAV1-EF1a-DIO-mCherry-scrmb-shRNAmir were injected into vS.

#### Behaviour

Following at least 3 weeks post-surgical recovery, animals (10 – 12 weeks old) were manually handled for at least 7 days before testing. During the last 3 days of habituation, empty plastic weighing boats were provided in the home cage to habituate the animals to these objects. During this time animals were also habituated to intraperitoneal (i.p.) injection, patch cord attachment (for photometry and optogenetic experiments) and the behavioural boxes as described below. All behavioural experiments were carried out in MEDPC sound-attenuating chambers containing behavioural boxes (21.59 x 18.08 x 12.7 cm) with blank walls. Video recordings were conducted with infrared cameras positioned above each chamber, and video was acquired at 15 or 25 Hz using Bonsai.^111^ The different frame rates were due to a change of PS4 cameras over the course of experiments, and this difference in frame rates did not affect the resolution of capturing naturalistic behaviour given the relatively slower time course of evolving behaviours during feeding. All experiments were performed consistently during the middle-to-end of the light cycle (from 2 pm to 7 pm) to control for circadian rhythm variables.

For all behavioural experiments, after acclimatisation to handling and the behavioural chambers, mice were habituated over the course of 2 to 3 days with three i.p. injections of 100 μl sterile phosphate-buffered saline (PBS) to habituate them to manual scruffing and i.p. injection. Following this, animals for photometry and optogenetics experiments were habituated to patch cord attachment for a 10-minute period, as described above. At the end of this habituation period, animals were given an i.p. injection of either ghrelin (2 mg/kg; Tocris) or vehicle control (phosphate-buffered saline, PBS; pH = 7.2). The order of the injections was counterbalanced across animals. The volume of the i.p. injection was fixed at 100 μl. Animals were allowed 15 mins to recover post-injection before the presentation of non-food and food objects. The day of ghrelin injection was selected randomly for each animal, and PBS and ghrelin injection days were spaced apart for a duration of at least 24 hours. After termination of each testing session, the amount of chow consumed during the 10 min presentation was weighed; any spillage of food was recovered and subsequently weighed.

For photometry experiments, the time of food or non-food presentation was noted down and used to manually synchronise the photometry recordings to the start of stimulus presentation. Photometry experiments with apparent failure in equipment or software acquisition, where calcium signals were not observable, or mice with misplacement of or damage caused by injections and implantations were excluded (10 in total).

For optogenetics experiments, light power at the end of each bilateral patch cord was 8 mW. Light delivery was given in a counterbalanced design, separated by at least 48 hours. On stimulation day, light was delivered at 20 Hz (5 ms on, 45 ms off), either throughout the duration of the experiment (Figures 5A–5C), or only when the mouse was investigating food (Figures 5D–5F), and analysis was performed on entire ON vs OFF sessions. Stimulation parameters were chosen to match previous studies of this circuit.^31^^,^^102^ One mouse was excluded due to mild, presumed seizure activity (cessation of normal behaviour, change in posture, slowness and lack of directed movement) upon light stimulation. For pharmacogenetics experiments, mice were injected with 0.5 mg / kg CNO (Hello Bio) dissolved in sterile PBS, or PBS as a control 30 minutes before the behavioural session, counterbalanced across days. Two mice were excluded due to misplaced injections. For antagonist experiments, 0.5 μg [D-lys3]-GHRP-6 (Tocris) in 0.5 μl sterile PBS was injected bilaterally into the vS of lightly anaesthetised mice 30 minutes before ghrelin injection. On counterbalanced control days, 0.5 μl of PBS was injected as a control.

#### Annotation of feeding behaviour

Feeding behaviour was analysed as a composite sequence of five simple, distinct and reproducible behaviours. These elemental behaviours were: Investigate (sniffing or tactile interaction with the object or food without eating), Eat (biting food or chewing movements close to food), Rear (standing on hindlegs while elevating head, can be supported on the walls i.e. thigmotaxis), Groom (licking/scratching of fur, limbs or tail, usually high-frequency movement) and Rest (motionless, usually in corner of box). These behaviours together are referred to as the Behavioural Satiety Sequence (BSS, ^66^). These features were manually scored offline using either Ethovision XT10 (Noldus) or BORIS.^112^ Where possible, manual scoring was conducted in a blinded fashion to experimental groups. For a subset of videos, two independent scorers conducted manual annotation of the behaviour videos to ensure reproducibility. Manual annotation of BSS behaviours from 10- minute videos spanning the food or non-food object presentation period were conducted at 15 or 25 Hz on a frame-by-frame basis. This manual annotation produced vectors of 0s and 1s, where 0 indicates the absence and 1 the presence of the BSS behaviour.

#### Analysis of feeding behaviour as a stochastic Markov process

Each behavioural dataset exists as a sequence of BSS behaviours. In other words, the behaviour for a given animal is described by a vector of BSS behaviours occurring over time. Although the total time spent engaging in one behaviour can be computed from this vector, additional information regarding an animal’s feeding strategy exists in the sequence of expression of each BSS behaviour.^12^ To analyse this sequential information in more detail, we analysed the annotated behavioural patterns for each mouse as a stochastic Markov process that defined the animal’s feeding strategy when presented with chow across different states of hunger. Specifically, a Markov chain is a vector of states that change as a function of time. In this case, the Markov chain is comprised of 5 states corresponding to the 5 BSS behaviours. These Markov chains are described fully by a transition matrix P, where the P*ij* term represents the transition probability from BSS behaviour *i* to *j*. As there are 5 BSS behaviours, P is a 5 × 5 transition matrix, where the rows represent the current BSS behaviour, the columns represent the BSS behaviour one-step ahead and the values in each row sums to 1. For display purposes, as non feeding behaviours showed little consistent, but independent alterations upon ghrelin injection, we constructed simpler, 3 x 3 transition matrices by combining non-feeding behaviours into a single state. For comparison, 5 x 5 matrix analysis is provided in Figure S1. To compute the empirical transition matrices for each animal, the frequency of each possible transition from behaviour *i* was calculated and normalised by the total number of behavioural transitions occurring from behaviour *i*. These transitions are assumed to be Markovian, which simplifies the calculation of the transition probability P(state = j | state = i). Specifically, the probability of transitioning from state i to state j is dependent only on the current state i and not on states preceding state i. In the case an animal did not perform a particular behaviour (for example, in Figure 1, two mice did not eat in the PBS condition), the transition probability from that state can be either scored as zero, or NaN. In this study we chose to keep these transitions as zero, as we felt this more accurately reflects the data, but note that this results in some rows of the transition matrix not summing to 1. Irrespective of this decision, there was no change in the quantitative comparisons presented in the paper. For each animal, there were two transition matrices, *P*_PBS_ and *P*_Ghrelin_. Importantly, these Markov chain vectors disregarded information relating to duration, i.e. the time spent in engaging in one behaviour and the inter-event duration. In other words, by focusing on transitions between BSS behaviours, this analysis was conducted time-agnostically; this method has been shown to accurately capture moment-to-moment behavioural strategies under differing contexts^12^ by focusing on the transition probability from one behavioural bout to the next. For example, the vector [Investigate, Investigate, Eat, Groom] represents four distinct BSS bouts of variable length within and between bouts, but only the transitions between bouts were analysed. Note behavioural data in Figure 1 contains mice also utilised for photometry experiments in Figures 2 and 3.

#### Analysis of transition matrix similarity

To quantify and compare the transition matrices between different states of hunger, we used the cosine distance as a similarity metric between two matrices.^12^ Behavioural transition matrices for each mouse were linearised to produce 25-dimensional vectors, with each element representing a transition probability P*ij*. Thus, for a within-subject comparison of the similarity in the transition vector between the sated and hungry states, the angle or cosine similarity between the two transition vectors u and υ, i.e. Fed and Fasted state transition vectors respectively, was computed using the following formula^12^:

$$\cos\emptyset =\frac{u\cdot \upsilon }{∥u∥∥\upsilon ∥}$$

where the cosine distance is:

1−cos∅

The range of the cosine distance is bounded in the interval [0, 1]. Values of the cosine distance closer to 0 indicate a high similarity and a smaller angle between the two vectors, while values closer to 1 indicate low similarity and a large angle between the two vectors.

#### Clustering of BSS behavioural transition matrices

We first reduced the dimension of the 25-dimensional transition matrices using Fisher’s linear discriminant analysis (LDA). LDA identifies the subspace that maximises the discriminability between the experimental manipulations of hunger, i.e Fed, Fasted, PBS and Ghrelin, by maximising the ratio of the between-class over the within-class variability. The between- and within-class variability were scatter matrices SB and SW, respectively, where SB and SW are 25 × 25 matrices and the number of rows or columns corresponds to each behavioural transition probability. The target variable was the hunger state (labels of Fed, Fasted, PBS and Ghrelin). The projection matrix used to project the vectors to the first two LDA axes was solved by matrix factorisation using singular value decomposition (SVD) based on the *LinearDiscriminantAnalysis* function from the *sklearn* package.

For clustering analysis in the reduced LDA subspace, Gaussian mixture model (GMM) clustering was performed using the *GaussianMixture* function from the *sklearn.mixture* package. GMM was applied on the n × 2 matrix containing the transformed behavioural transition probabilities in the first two LDA subspaces, where n is the number of observations (60 observations) in the rows of the matrix, and LD1 and LD2 are the columns of the matrix. The parameters for GMM clustering were the following: 0.3 regularisation to the diagonal of the covariance matrix, full covariance matrix for each component and 1000 maximum iterations. The Gaussian component weights were initialised by k-means. To select the number of Gaussian components that best fit the data, the Bayesian information criterion (BIC) was calculated:

$$BIC=k\log\;n-2\log\hat{L}$$

where k is the number of model parameters, n is the observation number and $\hat{L}$ is the maximum likelihood of the model. The BIC value was calculated using the *GaussianMixture* function.

Finally, a supervised random forest classifier (100 estimators and using the Gini criterion) was used to assess the robustness of the clustering.^113^ The classifier was iteratively trained with increments of 3 randomly chosen observations (0 to 39 observations) from a total of 60 observations, and the classifier was used to predict cluster identity of the remaining observations. Each training iteration was repeated 100 times with random sampling of observations to train the classifier (for example, training the classifier with 3 observations was repeated 100 times, with random sampling of 3 observations of the dataset). The accuracy of the classifier was computed as a function of the number of observations used to train the dataset.

#### Analysis of Ca2+ signals from fibre photometry

Measurement of calcium fluorescence signals was carried out as detailed previously.^59^^,^^114^ 470 nm and 405 nm LEDs were used as excitation sources, and the light amplitudes were modulated sinusoidally at 500 Hz and 210 Hz carrier frequencies, respectively. The excitation light was passed through excitation filters (for 470 nm and for 405 nm wavelengths), and a dichroic mirror to combine the two LEDs into a single beam. A 49/51 beam-splitter was used to split the beam into two independent excitation beams for simultaneous recording of two animals. The excitation light was coupled through a fibre collimation package into a fibre patch cord, and linked to a large core (200 μm), high NA (0.39) implant cannula (Thorlabs). Emitted fluorescence signals were collected through the same fibre. Fluorescence output signal was filtered through a GFP emission filter (transmission above 505 nm) and focused onto a femtowatt photoreceiver. The photoreceiver was sampled at 10 kHz, and each of the two LED signals was independently recovered using quadrature demodulation on a custom-written Labview software: this process involved using an LED channel as a signal reference, taking a 90-degree phase-shifted copy of this reference signal and multiplying these signals in quadrature. The multiplied signal was then low-pass filtered with a Butterworth filter (order = 3, cut-off frequency = 15 Hz). The hypotenuse was then computed using the square root of the sum of squares of the two channels. The result corresponds to the demodulated signal amplitude and was decimated to 500 Hz before storing to disk.

To correct for artefacts resulting from Ca^2+^-independent processes such as movement, the Ca^2+^-independent 405 nm isosbestic wavelength signal was scaled to the 470 nm wavelength. The coefficients for the scaling were computed through a least-squares linear regression between the 405 nm and 470 nm signal. This estimated motion (scaled 405 nm) signal was then subtracted from the 470 nm signal to obtain a pure Ca^2+^-dependent signal.

Calcium activity signals time-locked to the presentation of chow were extracted using the time of presentation manually determined from video recordings. The signal was decimated to 15 Hz, z-score normalised, filtered with a Gaussian filter (σ = 1.5) and baselined to the mean signal in the -50 to -10 seconds preceding the time of presentation of food or non-food object. For event-triggered analysis, the photometry signal was aligned to the onset of each behavioural event obtained from the manually scored behaviour. The behavioural events were clustered into bouts (defined as continuous engagement in the behaviour), and the onset of each bout was taken as the time point to align the photometry signal. A peri-event window of 20 s surrounding the onset of the behaviour was obtained for each signal, and the resulting signal was baselined to the time period from -10 to -7.5 seconds relative to the onset of each event. All trials obtained for an animal were averaged to obtain a nested average event-triggered signal; these signals were then averaged across mice to obtain the population event-triggered average. For experiments in Figures S3K–S3M, ghrelin-treated mice investigated the non-food item more frequently than PBS-treated mice, and so for comparison only the first 3 investigative bouts in each condition were analysed to ensure fair comparison. Due to the stochastic nature of emitting a given behaviour, not all behaviours were present in all animals. To avoid including the same behavioural events across multiple event triggered averages, only events separated by more than 5s were included. Animals displayed Investigative behaviour consistently in all internal states of hunger, but the proportion of animals showing Eat, Groom, Rest and Rear behaviours were variable.

#### Linear encoding model relating behaviour to neural activity

To quantify the contribution of each BSS behaviour to neural activity, a multiple regression model was used. The linear model was constructed using the Python package *sklearn*, with the z-scored baselined photometry signal as the dependent variable, and a regressor matrix of BSS behavioural arrays as independent variables. The regressor matrix contained 27 regressors in total: 5 behavioural regressors (Investigate, Eat, Rear, Groom and Rest), 20 behavioural transition regressors (for example, Investigate → Eat), a manual presentation regressor and a velocity regressor. The 5 behavioural regressors were coded as pulses of 0s and 1s, where 1s indicate the engagement in a BSS behaviour and 0s the absence of engagement. The 20 transition regressors were included to account for any possible contribution of behavioural transitions to the photometry signal, and were derived as follows: a Markov chain vector of BSS behaviours was produced and any across-BSS transitions (e.g. Investigate → Eat, not Investigate → Investigate) occurring within 5 seconds was emitted as a temporal pulse of 1 at the onset of the next BSS behaviour. To account for temporal distortion of the behavioural transition period in the associated Ca^2+^ activity, an exponential function was first computed:

$$g\left(t\right)=Ae^{Bt}$$

where:

A=1

$$B=-\frac{\log\left(\frac{1}{A}\right)+1}{t_{1/2}}=\frac{1}{t_{1/2}}$$

where $t_{1/2}$is the half-life of the exponential function and set to 2 seconds. The transition regressor was convolved with the exponential function:

$$f\left(t\right)*g\left(t\right)=\int_{-\infty }^{\infty }f\left(\tau \right)g\left(t-\;\tau \right)d\tau$$

where f(t) is the transition regressor and g(t)is the exponential function. This produces a sharp peak to 1 and a decay rate of $t_{1/2}$. The exponential decay function was set to have a half-life of 2 s to approximate near-complete decay of the GCaMP6f signal. The presentation regressor was set to an exponential function with a peak time at presentation onset and a decay rate of 5 seconds to capture the salience of stimulus presentation. Finally, the velocity regressor was a continuous variable tracking the instantaneous velocity of the animal derived from position tracking using Ethovision. To handle periods of discontinuous tracking, the velocity data were pre-processed with a ceiling of 5 cm/s (to handle large jumps in tracking), smoothed with a rolling mean filter (window = 3 seconds), and imputed via linear interpolated to handle missing values.

The final linear encoding model was therefore the following:

$$y=\beta_{0}+\sum_{n=1}^{N_{B}}\beta_{n}^{B}+\sum_{n=1}^{N_{Tr}}\beta_{n}^{Tr}+\beta^{P}+\beta^{V}+\varepsilon$$

where y is the dFz in one animal, $\beta_{0}$ is the intercept (bias), ε is a Gaussian noise term, $N_{B}$ and $N_{Tr}$ are the numbers of the behavioural and transition regressors (5 and 20, respectively), $\beta^{B}$, $\beta^{Tr}$, $\beta^{P}$ and $\beta^{V}$ are the beta weights for the behavioural, transition, presentation and velocity regressors, respectively. Specifically, the beta weights β^B^ can be interpreted as the isolated, average neural response to engagement in that BSS behaviour. The crucial aspect of the linear encoding model is the simultaneous inclusion of possible confounding variables, for example, behavioural transitions and velocity, which may each contaminate the neural response. The linear model thus aims to statistically disambiguate the neural response to BSS behaviour engagement from other events in close temporal proximity.

The linear model was fit using ridge regression, a version of the ordinary least- squares regression that penalises the size of the estimated β coefficients by L2 regularisation. This ensures that the β weights were constrained to avoid overfitting, and the penalty term α adjusts the degree of shrinkage of the β weights. Prior to fitting, the dataset was split into an 80% training set to estimate the β weights and 20% test set for evaluating the model predictions. On this training dataset, a nested cross-validation procedure was used: first, the training dataset was split into 5 folds for evaluation. For each fold, the α hyperparameter was tuned using leave-one-out cross-validation (GCV). GCV works analogously to a grid search by exploring the alpha parameter space, and selecting the α value that maximises the prediction accuracy of the model; the values of α tested were 10^−3^, 10^−2^, 10^−1^, 10^0^ and 10^1^, using the function RidgeCV on Python’s sklearn package. The values of α did not differ significantly between the PBS and Ghrelin conditions. The predictor matrix was normalised by subtracting the predictor matrix by its mean and dividing by the L2 norm of the matrix, using the function RidgeCV. The β weights were computed analytically using the following formula:

$$\beta ={\left({Χ}^{T}Χ+\alpha Ι\right)}^{-1}\left({Χ}^{T}y\right)$$

where Χ is the predictor matrix, α is the ridge penalty term, Ι is the identity matrix and y is the observed dFz. Once fitted, the performance of the linear encoding model was assessed by using the independent test set to compute the explained variance (5-fold, cross-validated R2) value, or the coefficient of determination, defined as:

$$R^{2}=1-\frac{u}{v}=1-\frac{\sum_{i}{\left(y_{i}-\hat{y}\right)}^{2}}{\sum_{i}{\left(y_{i}-\bar{y}\right)}^{2}}$$

where u is residual sum of squares, v is the total sum of squares, $y_{i}$ is the photometry signal at index i, $\hat{y}$is the predicted photometry signal at index i and $\bar{y}$ is the mean amplitude of the photometry signal in the test set. Linear models were estimated separately for data from individual animals.

#### Electrophysiology

Hippocampal recordings were studied in acute transverse slices as described previously.^57^^,^^58^^,^^59^ Mice were anaesthetized with a lethal dose of ketamine and xylazine, and perfused intracardially with ice-cold external solution containing (in mM): 190 sucrose, 25 glucose, 10 NaCl, 25 NaHCO3, 1.2 NaH2PO4, 2.5 KCl, 1 Na+ ascorbate, 2 Na+ pyruvate, 7 MgCl2 and 0.5 CaCl2, bubbled with 95% O2 and 5% CO2. Slices (400 μm thick) were cut in this solution and then transferred to artificial cerebrospinal fluid (aCSF) containing (in mM): 125 NaCl, 22.5 glucose, 25 NaHCO3, 1.25 NaH2PO4, 2.5 KCl, 1 Na+ ascorbate, 3 Na+ pyruvate, 1 MgCl2 and 2 CaCl2, bubbled with 95% O2 and 5% CO2. After 30 min at 35 °C, slices were stored for 30 min at 24 °C. All experiments were conducted at room temperature (22–24 °C). All chemicals were from Sigma, Hello Bio or Tocris.

Whole-cell recordings were performed on retrogradely labelled hippocampal pyramidal neurons with retrobeads visualised by their fluorescent cell bodies and targeted with Dodt contrast microscopy. For sequential paired recordings, neighbouring neurons were identified using a 40x objective at the same depth into the slice. The recording order of neuron pairs was alternated to avoid complications due to rundown. Borosilicate recording pipettes (3 – 5 M) were filled with different internal solutions depending on the experiment. For electrical stimulation experiments a Cs-gluconate based internal was used containing (in mM): 135 Gluconic acid, 10 HEPES, 7 KCl, 10 Na-phosphocreatine, 4 MgATP, 0.4 NaGTP, 10 TEA and 2 QX-314. Excitatory and inhibitory currents were electrically isolated by setting the holding potential at -70 mV (excitation) and 0 mV (inhibition) and recording in the presence of APV (50 μM). Alternatively, to record inhibitory miniature currents at -70 mV we used a high chloride internal (in mM): 135 CsCl, 10 HEPES, 7 KCl, 10 Na-phosphocreatine, 10 EGTA, 4 MgATP, 0.3 NaGTP, 10 TEA and 2 QX-314 in the presence of APV (50 μM), NBQX (10 μM) and TTX (1 μM) to block synaptic excitation and spontaneous IPSCs. Note for inhibitory mIPSC experiments (Figures 4E–4J), electrophysiological recording was only possible for vS-NAc neurons but not vS-LH neurons in two ghrelin-injected animals as there was poor retrograde labelling of vS-LH neurons (i.e. vS-NAc, n = 37 neurons from 5 animals; vS-LH, n = 21 neurons from 3 animals). Recordings were made using a Multiclamp 700B amplifier, with electrical signals filtered at 4 kHz and sampled at 10 kHz.

#### Validation of shRNAmiR-mediated GHSR1a knockdown

Mouse GHSR1a receptor was expressed with an N-terminal FLAG tag using a vector purchased from Origene (Cat# MR226073). For expression in HEK cells, shRNAi sequences were expressed from a pcDNA6.2-EmGFP-mir9 vector gifted by Lynn Hudson (RRID:Addgene_22741).^115^ HEK293T cells were cultured in DMEM supplemented with GlutaMAX, 10 % FBS, and 100 U/mL penicillin/100 μg/mL streptomycin in a humidified incubator at 37 °C supplemented with 5 % CO2 (all reagents supplied by Thermo Scientific). Cells were transfected in 6-well plates using 1 μg total DNA (0.2 μg GHSR1a vector, 0.8 μg shRNAmiR vector) and 4 μL Lipofectamine 2000 (Thermo Scientific) per well. Cells were collected three days after transfection and lysed using Dounce homogenization in μL lysis buffer (150 mM NaCl, 50 mM Tris pH 8.0, 0.5 % sodium deoxycholate, 0.1 % sodium dodecyl sulfate, 1 % Igepal CA630, 5 mM EDTA, 1 mM PMSF, 1 complete protease inhibitor tablet/100 mL). Following centrifugation at 21,130 x g for 20 min, proteins in each clarified lysate were de-glycosylated by addition of PNGaseF at 0.01 μg/μL for 3 hours at 37 °C. Proteins were denatured in LDS for 10 min at 60 °C before SDS-PAGE with NuPAGE 4-12 % BisTris gels (Thermo Fischer Scientific). FLAG-GHSR1a was detected using mouse monoclonal anti-FLAG antibody directly conjugated to HRP (1 in 1000 dilution, Sigma-Aldrich Cat# A8592-.2MG) whereas the loading control GAPDH was detected using mouse monoclonal anti-GAPDH antibody (1 in 5000 dilution, Bio-Rad Cat# VMA00046) coupled to goat polyclonal anti-mouse HRP secondary antibody (1 in 2500 dilution, Thermo Scientific Cat# 32230) Densitometry was performed in ImageJ (NIH, RRID:SCR_003070) with GHSR1a intensity in each sample normalized against the loading control GAPDH.

#### Histology

Mice were perfused with 4% PFA (wt / vol) in PBS, pH 7.4, and the brains dissected and postfixed overnight at 4 °C as previously described.^57^^,^^58^^,^^59^ In summary, 70 μm thick slices were cut using a vibratome (Campden Instruments) in either the transverse or coronal planes. Slices were mounted on Superfrost glass slides with ProLong Gold antifade mounting medium (Molecular Probes). NucBlue was included to label gross anatomy. Imaging was carried out with a Zeiss Axio Scan Z1, using a 10x air immersion lens and standard filter sets for excitation/emission at 365-445/50 nm, 470/40-525/50 nm, 545/25-605/70 nm and 640/30-690/50 nm. Raw images were exported using Zen software (Zeiss) and analyzed with FIJI. Images of large sections presented in figures are composite images made by stitching together multiple, tiled fields of view using Zen software.

#### Statistical analyses

All statistics were calculated using the Python packages *scipy, pingouin* and *statsmodels*. Summary data are reported throughout the figures as boxplots, which show the median, 75th and 95th percentile as bar, box and whiskers respectively. Individual data points are also superimposed to aid visualisation of variance. Example physiology and imaging traces are represented as the mean +/- s.e.m across experiments. For the majority of analyses presented, normality of data distributions was determined by visual inspection of the data points. All data were analysed using statistical tests described in the statistical summary. Correction for multiple comparisons was conducted using the Benjamini-Hochberg method, unless otherwise stated. The alpha level was defined as 0.05. No power analysis was run to determine sample size a priori. The sample sizes chosen are similar to those used in previous publications. Throughout the figures and text, the ^∗^ symbol represents p < 0.05, unless otherwise stated, and n.s. stands for not significant. Animals were randomly assigned to a virus cohort (e.g. ChR2 versus GFP), and as far as possible, littermates testing each variable of interest (e.g. GFP control versus ChR2) were present in each cohort to control for experiment-to-experiment variability. Where possible the experimenter was blinded to each mouse’s virus assignment when the experiment was performed. This was sometimes not possible due to e.g. the presence of the injection site in the recorded slice.

## Supplemental information

Document S1. Figures S1–S7

Table S1. Statistical summary, related to Figures 1, 2, 3, 4, 5, 6, and 7 Summary of statistical tests described in main and supplemental figures.

Document S2. Article plus supplemental information
