# Supplementary material for: Internal-state-dependent control of feeding behavior via hippocampal ghrelin signaling
Source: Neuron. Author manuscript; Available in PMC 2025 Nov 25. (PMC12614488; doi:10.1016/j.neuron.2023.10.016)
Supplement: Supplementary Materials [file EMS210472-supplement-Supplementary_Materials.zip › 1-s2.0-S0896627323007973-mmc1.pdf]

Neuron, Volume *112*

Supplemental information

Internal-state-dependent control of feeding behavior via hippocampal ghrelin signaling

Ryan W.S. Wee, Karyna Mishchanchuk, Rawan AlSubaie, Timothy W. Church, Matthew G. Gold, and Andrew F. MacAskill

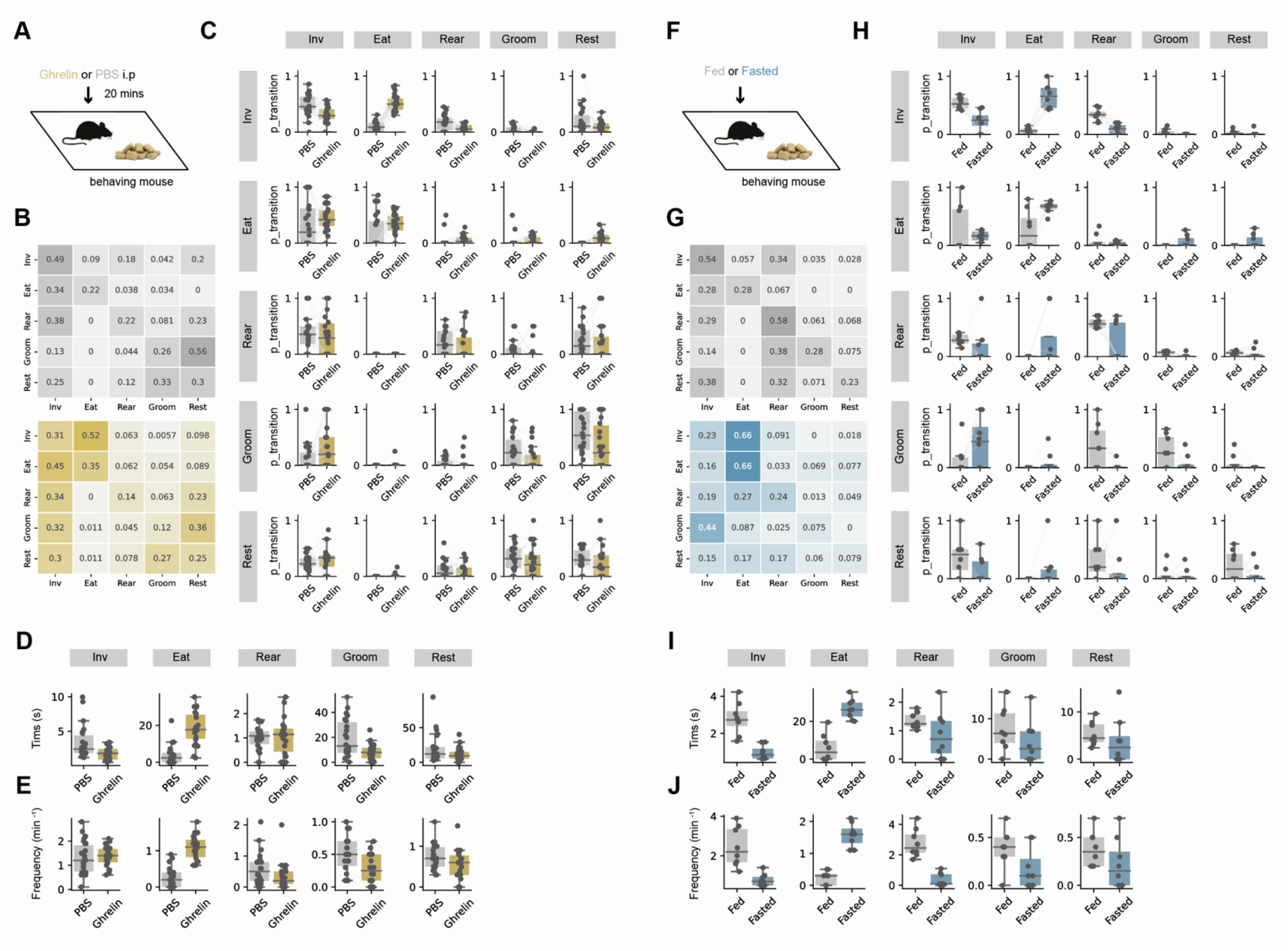

Supplementary Figure 1 | Expanded dataset for behaviour analysis. Related to Figure 1.

Supplementary Figure 1 | Expanded dataset for behaviour analysis. Related to Figure 1.

A. Top, schematic of experiment. Mouse was injected with either ghrelin or control PBS before exploring a well habituated chamber containing familiar chow for 10 min. Bottom, ghrelin administration (gold) increases chow consumption compared to PBS control (grey).

B,C. Full, 5-state Markov analysis of feeding behaviour during ten-minute session as shown in main figure, but displayed as box plots with individual mice.

D. Time spent engaging in each behaviour during ten-minute session.

E. Frequency of engaging in each behaviour during ten-minute session.

F-J. As in **A-E,** but for Fed and Fasted mice.

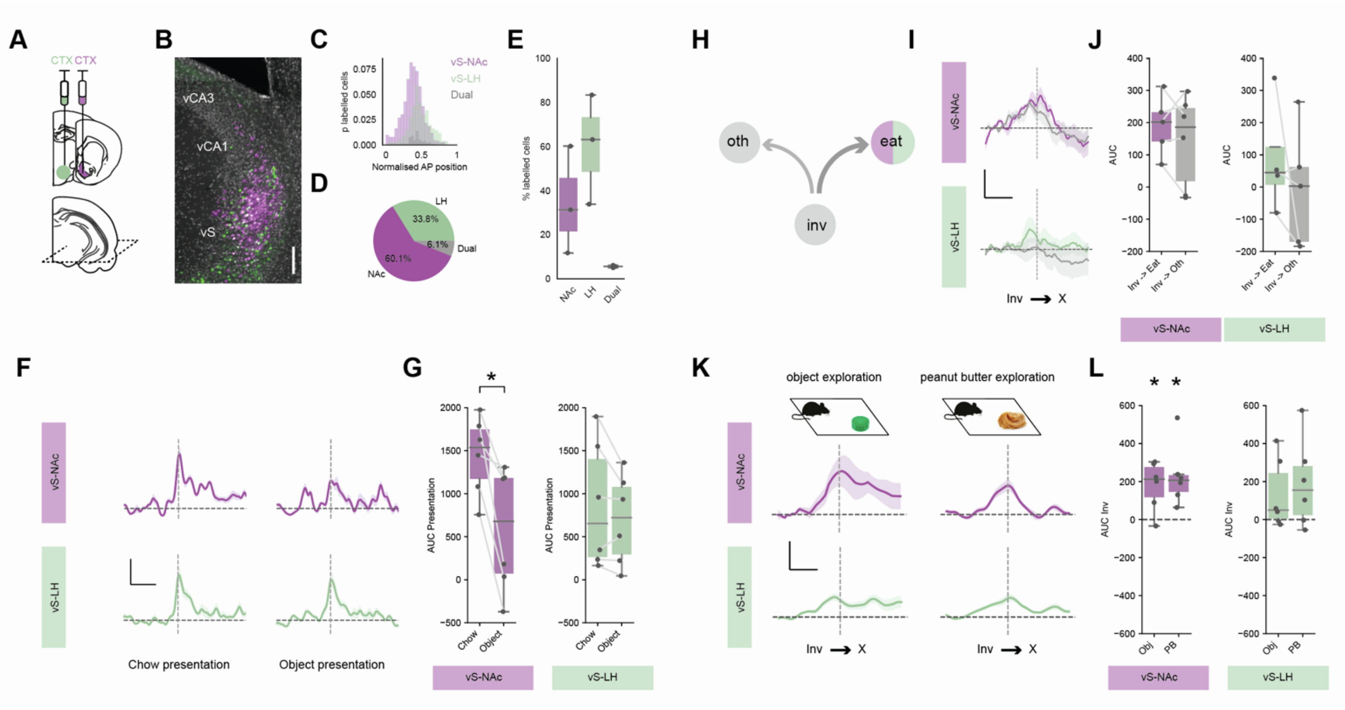

Supplementary Figure 2 | Additional anatomy and imaging analysis for vS-NAc and vS-LH neurons. Related to Figure 2.

Supplementary Figure 2 | Additional anatomy and imaging analysis for vS-NAc and vS-LH neurons. Related to Figure 2.

A. Schematic of injection of the retrograde tracer cholera toxin β conjugated to two different fluorophores into NAc and LH.

B. Example section showing retrogradely labelled neurons in vS. Scale bar = 100 μm.

C. Distribution of single and double labelled neurons along the proximal : distal axis of vS.

D,E. Proportion of double single and double labelled neurons for example (**D**), and across 3 experiments (**E**).

F. vS-NAc and vS-LH activity in response to presentation of Chow and non food object. Scale bar = 1zF, 50 s.

G. Summary of activity in vS-NAc (purple), and vS-LH (green) neurons. Note consistent vS-LH response to presentation.

H. Schematic showing activity around investigation is split into bouts followed by either eating, or no eating.

I,J. Activity of vS-NAc (purple) and vS-LH (green) neurons around investigation. Scale bar = 0.5 zF, 5 s. Note that for both vS-NAc and vS-LH neurons, activity is not different across investigative bouts followed by eating or not followed by eating.

K. Activity of vS-NAc (purple) and vS-LH (green) neurons around investigation of either a non-food object (left) or peanut butter (right). Scale bar = 0.5 zF, 5 s.

L. Summary of activity, note similar anticipatory ramping activity in response to both non-food objects and chow in vS-NAc neurons.

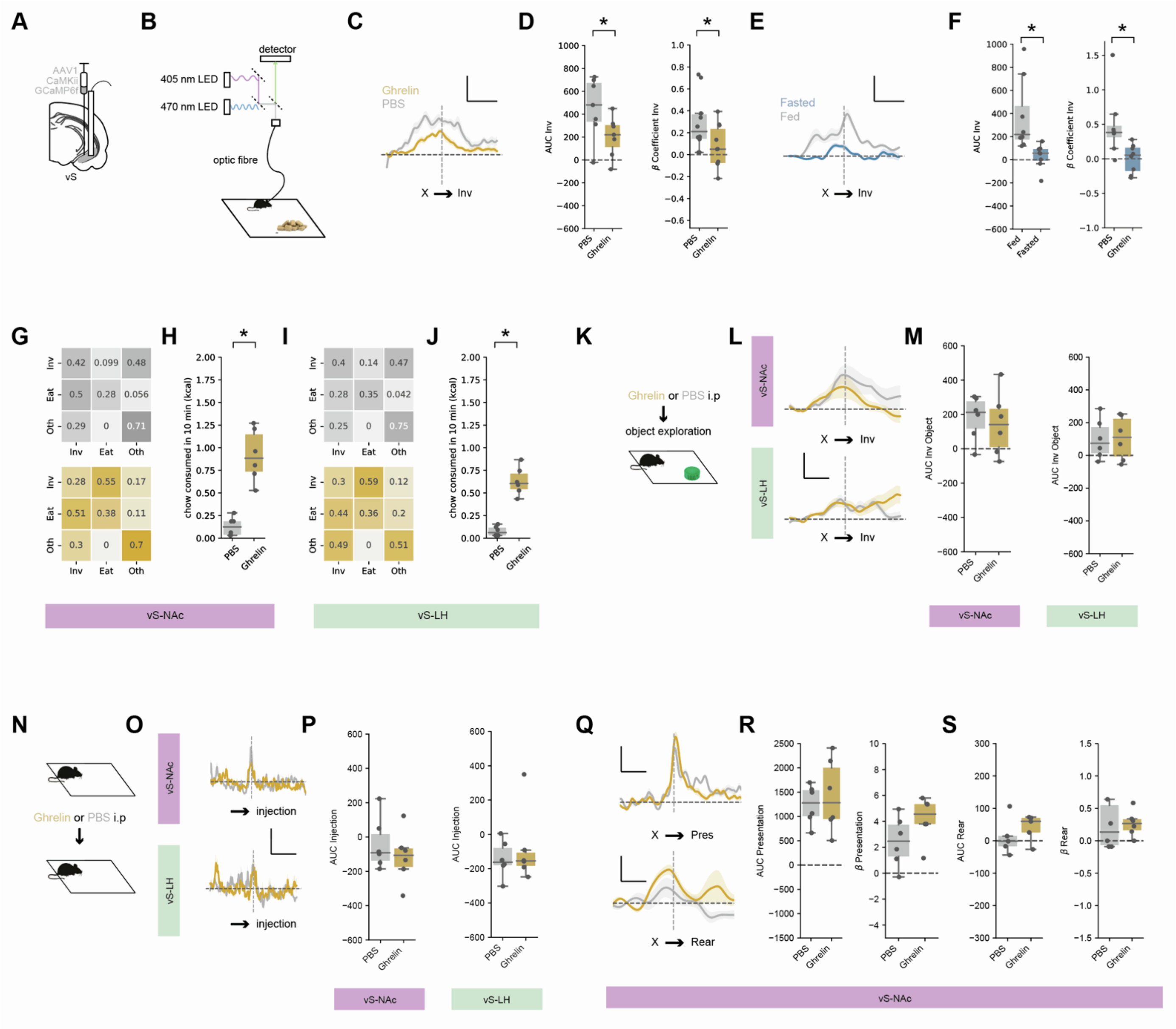

Supplementary Figure 3 | Additional analysis of the influence of ghrelin on vS, vS-NAc and vS-LH activity. Related to Figure 3.

Supplementary Figure 3 | Additional analysis of the influence of ghrelin on vS, vS-NAc and vS-LH activity. Related to Figure 3.

A,B. Schematic of GCaMP6f expression in vS to record activity via implanted optic fibre (**A**), and photometry optical path allowing recording in freely moving mice (**B**).

C. Average activity for vS neurons across all mice aligned to investigation, after injection of either PBS (grey) or ghrelin (gold). Note decrease in activity around investigation after ghrelin. Scale bar = 0.5 zF, 5 s.

D. Summary of activity around investigation for vS neurons after injection of either PBS (grey) or ghrelin (gold). *Left,* event-aligned AUC, *Right,* coefficients of a generalised liner model. In both analyses, note increase in activity is reduced after ghrelin.

E,F. As in **C,D** but in fed or overnight fasted animals. Note overnight fasting similarly reduces vS activity around investigation.

G. Markov analysis of feeding behaviour of mice used for vS-NAc recordings during ten-minute session. Transition matrix for PBS (top) and ghrelin (bottom) treated mice. Note that ghrelin increases the transition from investigation to eating, with minimal influence on other behavioural transitions.

H. Ghrelin administration (gold) increases chow consumption compared to PBS control (grey).

I,J. As in (**G,H**) but for vS-LH neurons.

K. Schematic showing experiment where mice investigate a non-food object.

L. Average activity for vS-NAc (purple) and vS-LH (green) neurons across all mice aligned to investigation of non-food object, after injection of either PBS (grey) or ghrelin (gold). Scale bar = 0.5 zF, 5 s.

M. Summary of activity during investigation of non food object. Note similar activity around investigation after ghrelin.

N. Schematic showing experiment where vS-NAc and vS-LH activity is recorded over the course of i.p. injection of either PBS or ghrelin.

O. Average activity for vS-NAc (purple) and vS-LH (green) neurons across all mice aligned to injection of either PBS (grey) or ghrelin (gold). Scale bar = 1 zF, 2 min.

P. Summary of activity after i.p. injections. Note similar activity after injection of either PBS or ghrelin.

Q. Activity of vS-NAc neurons aligned to presentation of chow (top) or rearing (bottom), after injection of either PBS (grey) or ghrelin (gold). Scale bar = 1zF, 50 s (top), Scale bar = 0.5 zF, 5 s (bottom).

R,S. Summary of activity around presentation of chow (R) and rearing (S). Not that activity around both events does not decrease after ghrelin treatment.

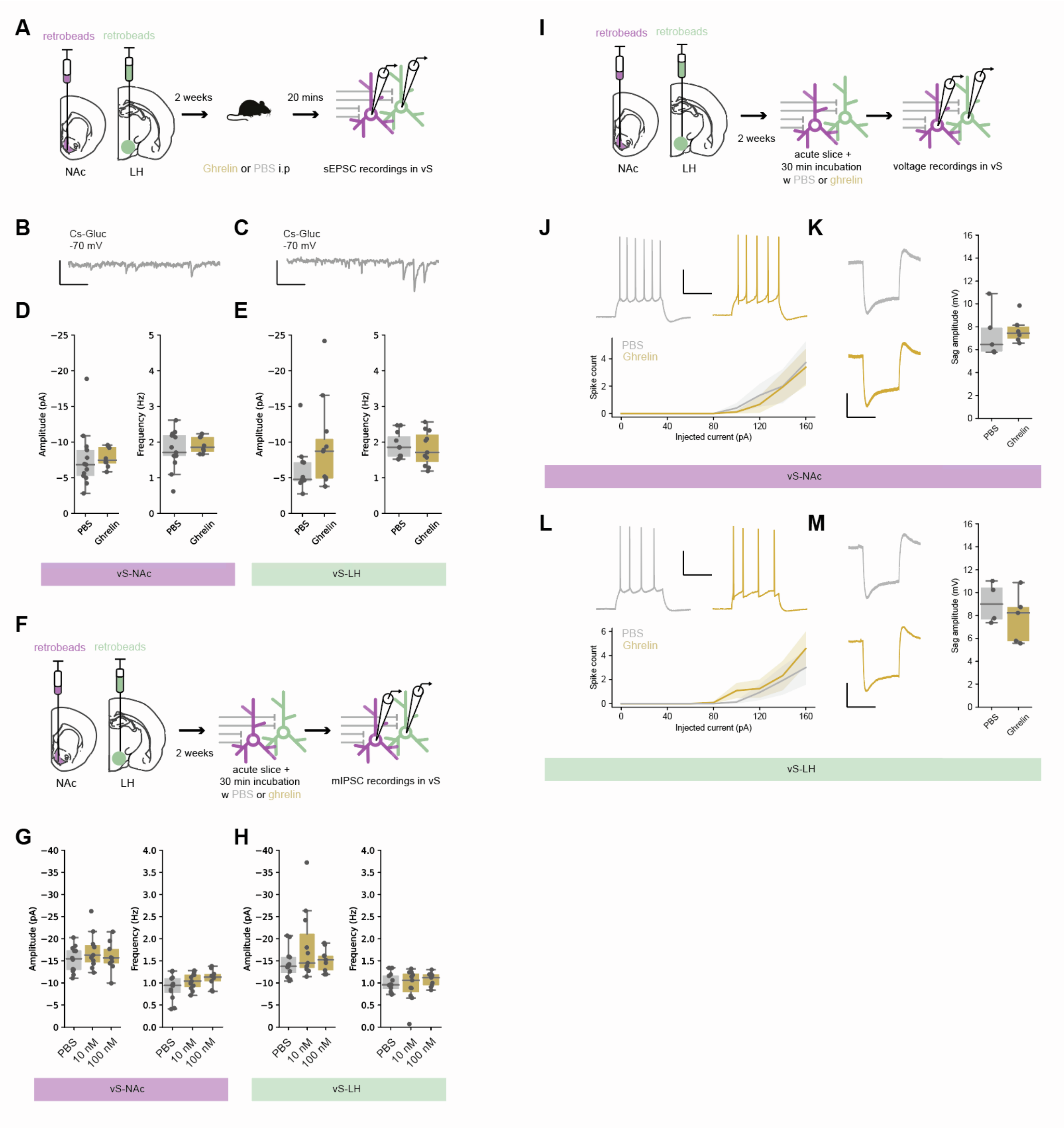

Supplementary Figure 4 | Behaviour of mice used for vS-NAc and vS-LH recordings is equivalent. Related to Figure 4.

Supplementary Figure 4 | Behaviour of mice used for vS-NAc and vS-LH recordings is equivalent. Related to Figure 4.

A. Schematic of retrobead injections allowing whole cell recordings of vS-NAc and vS-LH neurons. Two weeks after injections mice were treated with either ghrelin or PBS control, and acute slices were prepared 20 mins later.

B,C. Example traces containing isolated sEPSCs in vS-NAc (**B**) and vS-LH (**C**) neurons. Scale bar = 20 pA, 1s.

D,E. Summary of amplitude (left) and frequency (right) of sEPSCs in vS-NAc (**D**) and vS-LH (**E**) neurons. Note lack of consistent changes across any measures.

F. Schematic of retrobead injections allowing whole cell voltage clamp recordings of vS-NAc and vS-LH neurons. Two weeks after injections, acute slices were prepared and PBS or ghrelin (10 nM or 100 nM) was bath applied for 30 minutes before recordings.

G,H. Summary of amplitude (left) and frequency (right) of mIPSCs in vS-NAc (**G**) and vS-LH (**H**) neurons. Note lack of consistent changes across any measures.

I. Schematic of retrobead injections allowing whole cell current clamp recordings of vS-NAc and vS-LH neurons. Two weeks after injections, acute slices were prepared and PBS or ghrelin (100 nM) was bath applied for 30 minutes before recordings.

J. *Top,* Example spiking profile of vS-NAc neurons in response to a +160 pA current injection. Scale bar = 500 ms, 30 mV. *Bottom,* current input-spike output curves for vS-NAc after PBS or ghrelin incubation, demonstrating the number of action potentials as a function of injected current.

K. *Left,* example voltage responses of vS-NAc neurons in response to negative, -160 pA current injections Scale bar 500 ms, 10 mV. *Right,* Sag amplitude (proportional to *h*-current) after PBS or ghrelin incubation.

L,M. As in **J,K** but for vS-LH neurons.

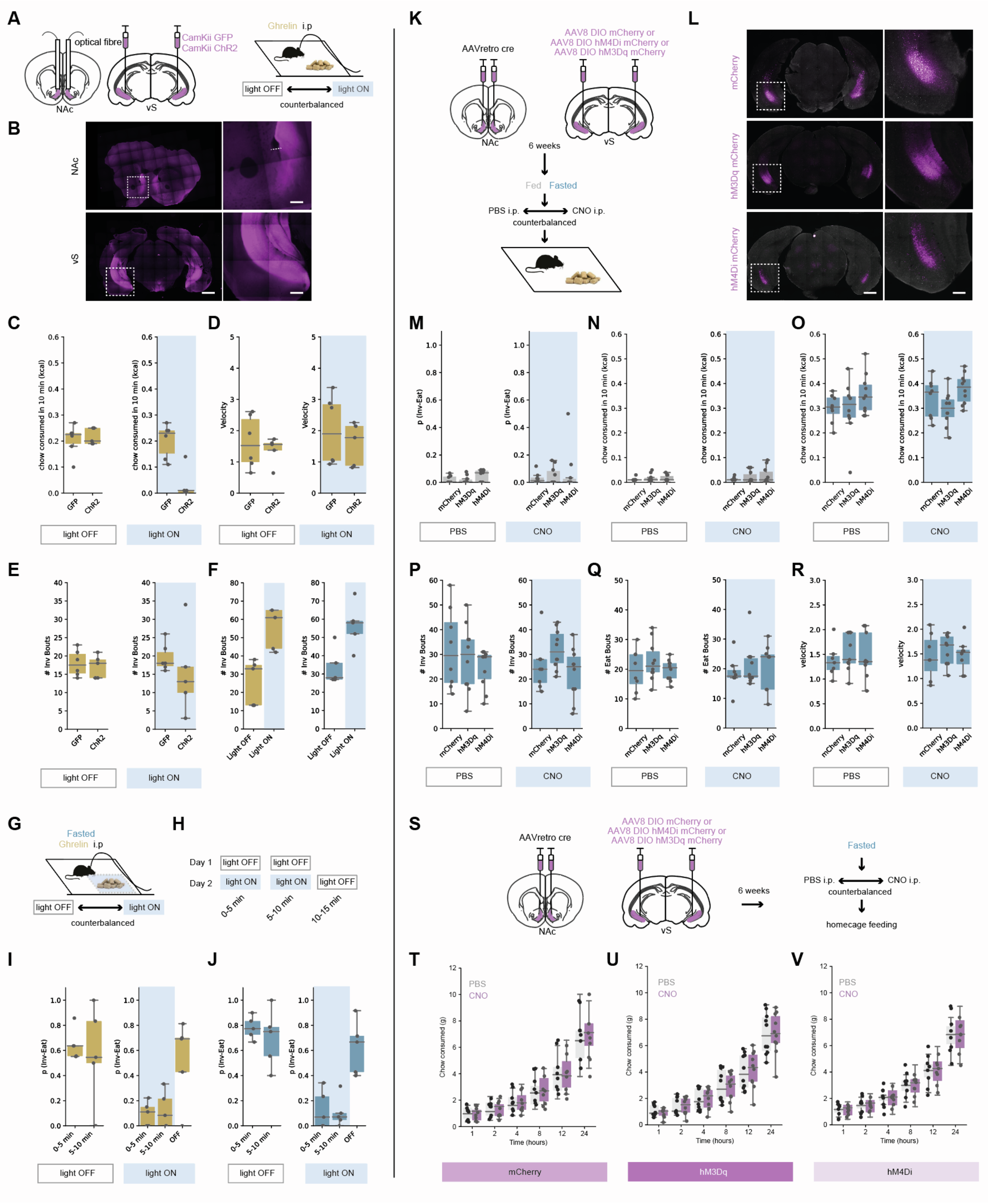

Supplementary Figure 5 | Additional analysis of vS-NAc activity manipulations. Related to Figure 5.

Supplementary Figure 5 | Additional analysis of vS-NAc activity manipulations. Related to Figure 5.

A. *Left,* Schematic of injections allowing optogenetic activation of vS-NAc neurons. *Right,* schematic of experiment. Activity of vS-NAc neurons was manipulated while mice explored a familiar arena containing chow after i.p. injection of ghrelin. For both GFP and ChR2 groups mice underwent the session twice, with 20 Hz light stimulation or no light stimulation in a counterbalanced order.

B. Example images showing expression ChR2 in vS (bottom) and axonal labelling in NAc, as well as fibre placement above NAc shell (top). Scale bar = 1mm (left), 100 µm (top right), 250 µm (bottom right).

C. Chow consumed during optogenetic experiments. Note vS-NAc activation results in almost complete cessation of consumption.

D. Average velocity (cm/s) of mice across each stimulation condition. Note velocity does not change as a result of vS-NAc stimulation.

E. Frequency of food investigation across each condition. Note that despite almost complete cessation of p(Inv->eat), number of investigative bouts is unchanged.

F. Frequency of food investigation in closed loop optogenetic experiments in ghrelin treated (left) and fasted (right) mice. Note that vS-NAc activation only around investigation, results in an increase in the number of food investigations.

G. Schematic of closed loop experimental design. Activity of vS-NAc neurons was manipulated while mice explored a familiar arena containing chow after i.p. injection of ghrelin, or overnight fasting.

H. Mice underwent the session twice, with 20 Hz light stimulation only when investigating food (Light ON), or no light stimulation (Light OFF) in a counterbalanced order. Sessions were split into 5 minutes epochs for subsequent analysis. Light ON sessions were followed by 5 minutes with no stimulation to investigate long term consequences of vS-NAc activation.

I,J. Summary of p(Inv->eat) on light OFF (left) and Light ON (right) sessions in ghrelin treated (**I**), and fasted (**J**) mice. Note stimulation resulted in a large decrease in p(Inv-eat), that was consistent across the session, and returned to baseline on cessation of light delivery.

K. *Top,* schematic of injections allowing pharmacogenetic activation and inactivation of vS-NAc neurons. *Bottom,* schematic of experiment. Activity of vS-NAc neurons was manipulated while overnight fasted mice explored a familiar arena. All mice underwent the session twice, 30 mins after i.p. injection of either PBS or CNO, counterbalanced across days.

L. Example images showing expression of mCherry, hM3D, and hM4D bilaterally in vS. Scale bar = 1 mm (left), 300 µm (right).

M, N. Summary of p(Inv->eat) (**M**) and chow consumption (**N**) in fed mice after PBS (left) or CNO (right) injection. Note there is no influence of vS activity manipulations in Fed mice.

O. Summary of chow consumed in fasted mice after PBS (left) or CNO (right) injection. Note that despite subtle trends, there is no influence of vS-NAc activity manipulations on chow consumption.

P-R. Summary of investigation frequency (**P**), eating frequency (**Q**), and velocity (**R**) in fasted mice after PBS (left) or CNO (right) injection. Note that despite subtle trends for potentially compensatory changes in investigation frequency, there is no influence of vS-NAc activity manipulations on any of these measures.

S. *Left,* schematic of injections allowing pharmacogenetic activation and inactivation of vS-NAc neurons. *Right,* schematic of experiment. Activity of vS-NAc neurons was manipulated while overnight fasted mice remained in their home cage, and chow consumption was measured at regular intervals. All mice underwent the session twice, after i.p. injection of either PBS or CNO, counterbalanced across days.

T-V. Summary of how chow consumption at intervals over 24 hours is influenced by vS-NAc activity manipulations. Note no effect of any condition.

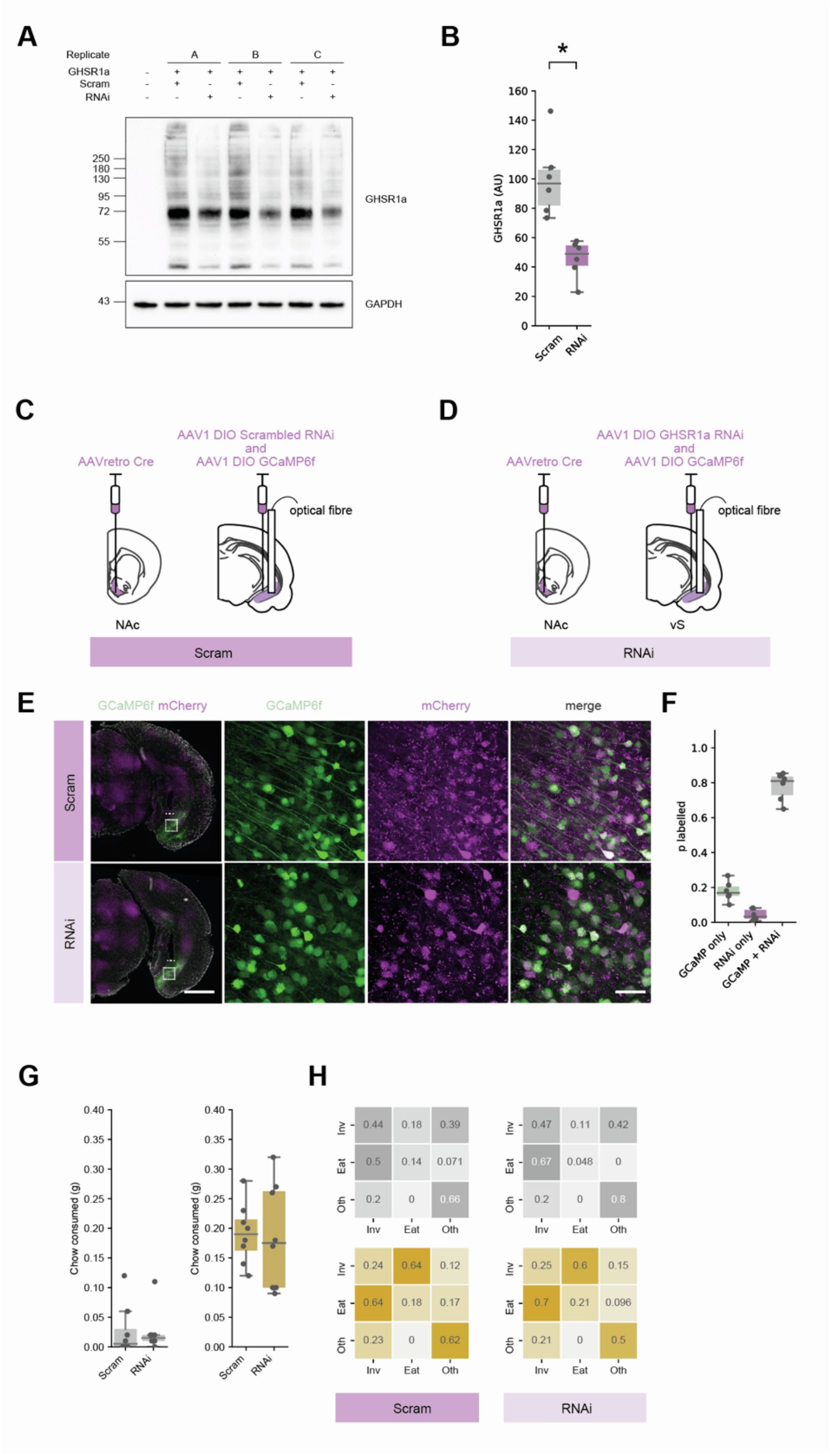

Supplementary Figure 6 | Characterisation of GHSR1a RNAi and demonstration that unilateral GHSR1a knockdown does not affect behaviour. Related to Figure 6.

Supplementary Figure 6 | Characterisation of GHSR1a RNAi and demonstration that unilateral GHSR1a knockdown does not affect behaviour. Related to Figure 6.

A. Example blot showing three replicates of expression of exogenous GHSR1a accompanied with scrambled control RNAi, or accompanied with GHSR1a RNAi. Note knockdown of GHSR1a after expression of GHSR1a RNAi compared to scrambled RNAi.

B. Quantification of GHSR1a knockdown across 6 replicates.

C,D. Schematic of injections allowing intersectional targeting of vS-NAc neurons with either scrambled control RNAi (**C**), or GHSR1a RNAi (**D**) as well as GCaMP6f and an optical fibre to allow photometry recordings.

E. Example images showing co-expression of GCaMP6f (green) with RNAi constructs (purple) in vS-NAc neurons after intersectional targeting, and fibre placement. Scale bar = 1 mm (left) 100 μm (right).

F. Quantification of vS neurons expressing GCaMP alone (green), RNAi alone (purple), or both (grey). Note the majority of neurons express both constructs.

G,H. Behavioural analysis showing no effect of unilateral GHSR1a RNAi expression of total chow consumed (**G**), or transition matrices (**H**). Note this lack of effect is in contrast to bilateral expression in **Figure 7**.

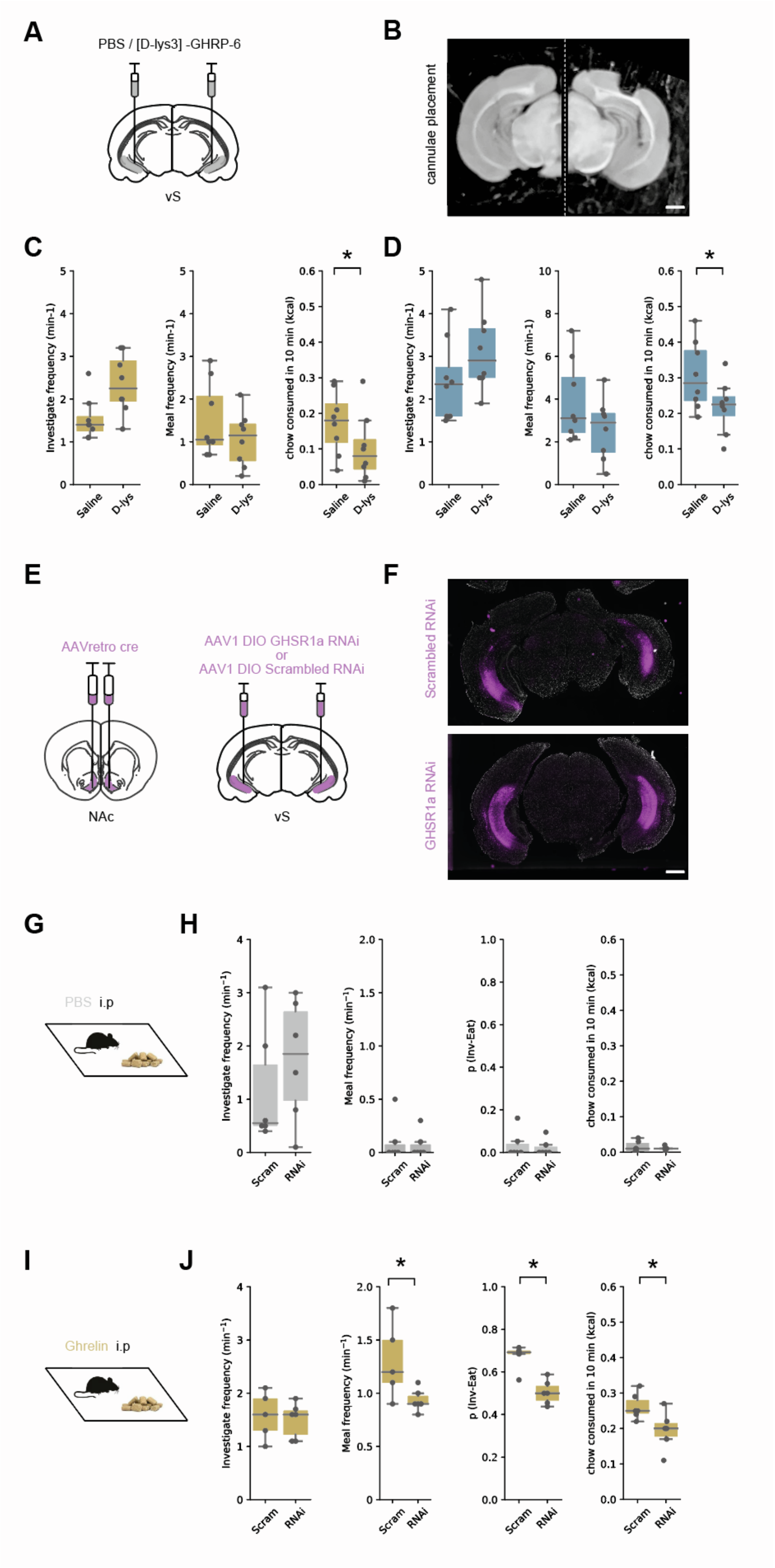

Supplementary Figure 7 | Additional analysis of behavioural effect of vS GHSR1a manipulation. Related to Figure 7.

Supplementary Figure 7 | Additional analysis of behavioural effect of vS GHSR1a manipulation. Related to Figure 7.

A, B. Schematic of injections into vS through implanted cannulae (**A**), and example image showing cannula placements bilaterally in vS (**B**). Note cannula placement site was in a different slice for each hemisphere and so image is a composite of two different slices (indicated by white dotted line). Scale bar = 1 mm.

C. Summary of frequency of investigation, frequency of eating and chow consumption in ghrelin treated animals injection with either PBS or D-Lys. Note that despite large influence on p(Inv->eat), there is a complex interaction between investigation and consumption. where increases in the frequency of investigation, mask changes in eating. similar to vS-NAc activity manipulations.

D. As in (**C**) but for fasted mice.

E,F. Schematic of injection strategy to express scrambled control or GHSR1a RNAi bilaterally in vS-NAc neurons (**E**), and example images showing expression bilaterally in vS (**F**). Scale bar = 1 mm.

G,H. Schematic of experiment (**G**) and summary of frequency of investigation, frequency of eating, p(Inv->eat) and chow consumption in Scrambled or GHSR1a RNAi expressing animals after PBS treatment. Note no effect of RNAi.

I,J, As in (**G,H**) but for ghrelin-treated animals. Note similar frequency of investigation of food, but reduced eating frequency, and reduced p(Inv->eat) in RNAi expressing animals.
